# Antiviral activity and mechanism of the antifungal drug, anidulafungin, suggesting its potential to promote treatment of viral diseases

**DOI:** 10.1186/s12916-022-02558-z

**Published:** 2022-10-21

**Authors:** Shu Shen, Yaxian Zhang, Zhiyun Yin, Qiong Zhu, Jingyuan Zhang, Tiantian Wang, Yaohui Fang, Xiaoli Wu, Yuan Bai, Shiyu Dai, Xijia Liu, Jiayin Jin, Shuang Tang, Jia Liu, Manli Wang, Yu Guo, Fei Deng

**Affiliations:** 1 State Key Laboratory of virology and National Virus Resource Center, Wuhan Institute of Virology, Chinese Academy of Sciences, Xiaohongshan 44#, Wuchang District, Wuhan, 430071 Hubei China; 2 State Key Laboratory of Medicinal Chemical Biology and College of Pharmacy, Nankai University, Tianjin, 300350 China; 3 College of Life Science, Nankai University, Tianjin, 300350 China

**Keywords:** Anidulafungin, Antiviral, SFTSV, SARS-CoV-2, Virus entry, Antifungal, Echinocandin

## Abstract

**Background:**

The severe fever with thrombocytopenia syndrome disease (SFTS), caused by the novel tick-borne SFTS virus (SFTSV), was listed among the top 10 priority infectious disease by World Health Organization due to the high fatality rate of 5–30% and the lack of effective antiviral drugs and vaccines and therefore raised the urgent need to develop effective anti-SFTSV drugs to improve disease treatment.

**Methods:**

The antiviral drugs to inhibit SFTSV infection were identified by screening the library containing 1340 FDA-approved drugs using the SFTSV infection assays in vitro. The inhibitory effect on virus entry and the process of clathrin-mediated endocytosis under different drug doses was evaluated based on infection assays by qRT-PCR to determine intracellular viral copies, by Western blot to characterize viral protein expression in cells, and by immunofluorescence assays (IFAs) to determine virus infection efficiencies. The therapeutic effect was investigated in type I interferon receptor defective A129 mice in vivo with SFTSV infection, from which lesions and infection in tissues caused by SFTSV infection were assessed by H&E staining and immunohistochemical analysis.

**Results:**

Six drugs were identified as exerting inhibitory effects against SFTSV infection, of which anidulafungin, an antifungal drug of the echinocandin family, has a strong inhibitory effect on SFTSV entry. It suppresses SFTSV internalization by impairing the late endosome maturation and decreasing virus fusion with the membrane. SFTSV-infected A129 mice had relieving symptoms, reduced tissue lesions, and improved disease outcomes following anidulafungin treatment. Moreover, anidulafungin exerts an antiviral effect in inhibiting the entry of other viruses including SARS-CoV-2, SFTSV-related Guertu virus and Heartland virus, Crimean-Congo hemorrhagic fever virus, Zika virus, and Herpes simplex virus 1.

**Conclusions:**

The results demonstrated that the antifungal drug, anidulafungin, could effectively inhibit virus infection by interfering with virus entry, suggesting it may be utilized for the clinical treatment of infectious viral diseases, in addition to its FDA-approved use as an antifungal. The findings also suggested to further evaluate the anti-viral effects of echinocandins and their clinical importance for patients with infection of viruses, which may promote therapeutic strategies as well as treatments and improve outcomes pertaining to various viral and fungal diseases.

**Supplementary Information:**

The online version contains supplementary material available at 10.1186/s12916-022-02558-z.

## Background

The severe fever with thrombocytopenia syndrome disease (SFTS) was first reported in 2010 in eastern China, with an initial fatality rate approximating 30%, and later in Japan, South Korea, and Vietnam [1–4]. Typical clinical manifestations of SFTS include high fever and thrombocytopenia, accompanied by gastrointestinal, respiratory, and neurologic symptoms as well as hemorrhagic manifestations, ranging from severe to fatal cases [5]. Records of the National Notifiable Disease Report System indicate that a total of 13,824 SFTS cases, including 713 deaths, were reported in China from 2010 to 2019, amounting to an average annual fatality rate of 5.2% [6]. The severe fever with thrombocytopenia syndrome virus (SFTSV), isolated from the sera of patients with acute infection, was identified as the causative pathogen of SFTS [1]. It is classified under the order *Bunyavirales*, family *Phenuiviridae*, and genus *Bandavirus*. SFTSV infections, which have been reported every month, mostly from April to October each year, show spatial and temporal distribution patterns that coincide with the time and location of tick activities [6]. The tick, *Haemaphysalis longicornis*, acts as the main reservoir and vector of SFTSV and transmits the virus to human and animal hosts via the process of blood feeding, thereby posing a threat to field workers [7]. Moreover, human-to-human transmission has been reported via direct contact with SFTSV-contaminated blood, fluids, or personal belongings of patients with SFTS [8, 9]. SFTS remains an important public health issue in China due to the high fatality rates and the lack of effective antiviral drugs for therapeutic purposes. To date, no specific antiviral drug has been approved for the treatment of SFTS. Generally, patients receive symptomatic and supportive therapy immediately after being diagnosed with SFTSV infection. The synthetic broad-spectrum antiviral drug, ribavirin, which has been approved for the treatment of several viral infections, such as Crimean-Congo hemorrhagic fever and Rift Valley fever, effectively inhibited SFTSV infection in vitro [10]. However, ribavirin therapy did not exert a significant effect on improving the clinical outcomes of SFTS, and common adverse events such as anemia and hyperamylasemia have been increasingly observed in patients receiving ribavirin therapy [11]. Thus, the need to improve clinical outcomes associated with SFTS and reduce fatality rates by developing effective anti-SFTSV drugs and therapy strategies may be regarded as vital.

SFTSV virions are sphere particles with a diameter of 80–100 nm [1]. The genome of SFTSV contains three RNA segments: L, M, and S, which encode the RNA-dependent RNA polymerase (RdRp), the glycoprotein (GP), and the nucleoprotein (NP) and the nonstructural protein (NSs), respectively [1]. The infection cycle of SFTSV in cells included multiple steps. SFTSV GP, which could be cleaved into Gn and Gc, mediates virus entry by binding to cellular receptors and inducing virus fusion with the cell membrane [12]. SFTSV enters into cells through the clathrin-mediated endocytosis pathway, and virus fusion occurs within the late endosome, which allows the release of the viral ribonucleoprotein (RNP) complexes into the cytoplasm [13]. SFTSV genome RNA replication occurs in the cytosol, which is initiated de novo by SFTSV RdRp and then synthesizes the antigenomic complementary RNA (cRNA), genomic viral RNA (vRNA), and capped, mostly non-polyadenylated viral mRNA [14]. So far, details on the process of SFTSV assembly and virion release were not clearly illustrated. Investigation of Uukunniemi virus and Rift Valley fever virus genetically related to SFTSV revealed that GP translation occurs at the rough endoplasmic reticulum (ER), which would be cleaved into Gn and Gc and processed with post-translational modifications. Correctly folded Gn and Gc are transported to the Golgi apparatus and associate with viral RNPs. New viral particles would form by budding into the Golgi apparatus and are released by exocytosis via transporting the virion-containing vesicles to the plasma membrane [15]. Antiviral strategies and specific drugs could be designed and developed by targeting these steps so as to inhibit SFTSV infection and prevent the virus spreading.

In this study, we screened an FDA-approved drug library for chemicals against SFTSV infection, and six drugs were identified as effective inhibitors of the entire SFTSV infection process in vitro. Of these, anidulafungin, the anti-fungal drug belonging to the echinocandin family, was found to inhibit SFTSV infection at the virus entry stage. The mechanisms underlying anidulafungin inhibiting SFTSV entry were further investigated based on infection assays, and the inhibitory effect was further verified in vivo using the SFTSV-infected mouse model. Furthermore, it was revealed that the antiviral activity of anidulafungin inhibits the entry process of other viruses more than SFTSV. The results revealed the antiviral activity and mechanism of anidulafungin, which is more than our knowledge about its antifungal activity by suppressing bacterial cell wall formation. The findings may promote the therapeutic strategies as well as treatments and improve outcomes pertaining to various viral and fungal diseases, regarding the use of anidulafungin as well as other echinocandins at clinical levels.

## Methods

### Cells and viruses

African green monkey kidney cells (Vero; ATCC: CCL-81) and human embryonic kidney cells (HEK-293; ATCC: CRL-1573) were maintained in Eagle’s Minimum Essential Medium (EMEM, NewZongke, Wuhan, China) supplemented with 10% fetal bovine serum (FBS; Gibco, Grand Island, NY, USA). Human hepatocarcinoma cells (Huh7), obtained from the National Virus Resource Center (NVRC number: IVCAS 9.005), were grown in Dulbecco’s modified Eagle’s medium (DMEM, NewZongke) supplemented with 10% FBS. The cell lines were tested negative for mycoplasma contamination using the commercial EZ-PCR Mycoplasma test kit (Biological Industries, Kibbutz Beit Haemek, Israel). All viruses used in this study, including the SFTSV strain WCH-2011/HN/China/isolate97 (WCH) [16], Guertu virus (GTV) strain DXM [17], Crimean-Congo hemorrhagic fever virus (CCHFV) strain YL16070 [18], and severe acute respiratory syndrome coronavirus 2 (SARS-CoV-2) strain WIV04 [19], were those preserved in the National Virus Resource Center (NVRC number: IVCAS 6.6088, IVCAS 6.6311, IVCAS 6.6106, IVCAS 6.6329, and IVCAS 6.7512, respectively). The herpes simplex virus 1 (HSV-1), strain MP, was obtained from the American Type Culture Collection (ATCC; VR-1383). Zika virus (ZIKV, strain FSS 13025) and Heartland virus (HRTV, strain MO-4) were obtained from the World Reference Center for Emerging Viruses and Arboviruses (University of Texas Medical Branch). Proliferation and in vitro experiments were conducted in the biosafety level 2 lab for SFTSV, GTV, HRTV, ZIKV, and HSV-1, and in the biosafety level 3 lab for CCHFV and SARS-CoV-2, according to the list of pathogenic microorganisms transmitted to humans issued by the National Health Commission of China.

### Antibodies and reagents

In-house prepared polyclonal antibodies, including anti-SFTSV NP [20], anti-SFTSV Gn [17], anti-SFTSV Gc (Additional file 1: Supporting information), anti-SFTSV NSs [21], anti-HRTV NP [22], anti-CCHFV NP [18], and anti-SARS-CoV-2 NP [23], were used to detect viral protein expression in cells via Western blotting or immunofluorescence assays. Mouse monoclonal antibodies, including the clone D1-4G2-4-15, specific to the flavivirus group antigen (NativeAntigen, Oxford, UK), and m27f, targeting HSV-1 and HSV-2 glycoprotein D (gD) [24], were used to detect ZIKV and HSV-1 infection in cells by immunofluorescence analysis (IFAs), respectively. Anti-β-actin monoclonal antibody (Sangon Biotech, Shanghai, China) was used as a control to detect cellular protein expression. HRP-conjugated Affinipure goat anti-rabbit IgG (H+L) (Proteintech Group, Wuhan, China) and HRP-conjugated goat anti-mouse IgG (H+L) (Proteintech Group) were used as secondary antibodies for Western blotting, while goat anti-mouse IgG H&L (Alexa Fluor® 488) (Abcam, ab150113, Cambridge, UK) and goat anti-rabbit IgG H&L (Alexa Fluor® 488) (Abcam, ab150077) were used as secondary antibodies.

Chemical compounds, including anidulafungin, micafungin, and caspofungin, were purchased from Selleck Chemicals (Houston, TX, USA). Endocytosis inhibitors, chlorpromazine (CPZ), nystatin, and p21-activated kinase inhibitor III (IPA3), and the vehicle, dimethyl sulfoxide (DMSO), were purchased from Sigma-Aldrich (St. Louis, USA). LysoSensor yellow/blue DND-160 and Rhodamine B chloride (R18) were purchased from Invitrogen (Carlsbad, USA). Favipiravir was kindly provided by Prof. Yu Guo from Nankai University, and 4′, 6-diamidine-2-phenylindole dihydrochloride (DAPI), used for staining cell nuclei, was obtained from Beyotime (Shanghai, China).

### Screening of FDA-approved drug library

As previously described, we screened a library of 1430 FDA-approved drugs (Selleck, https://www.selleckchem.com/screening/fdaapproved-drug-library.html) for anti-SFTSV activity, using SFTSV infection assays with slight modifications [25]. Briefly, Vero cells seeded in 96-well plates were treated with each drug or vehicle (DMSO) with favipiravir as the positive control for 1 h, and then inoculated with SFTSV at a multiplicity of infection (MOI) of 1. The cells were fixed at 24 h post-infection (p.i.), following which infected cells were detected via IFA using an antibody against the SFTSV NP, as previously described [26]. Images were captured, and the number of infected cells and the total number of cells in each well were measured using the Operetta CLSTM high-throughput system (PerkinElmer, Waltham, MA, USA). The percentage of inhibition following each drug treatment was determined by calculating the number of non-infected cells relative to the total number of cells. To validate the anti-SFTSV activity of these 48 drugs, Vero cells were treated with each drug at serially diluted concentrations and infected with SFTSV (MOI = 1). An IFA assay was performed at 24 h p.i., and percentage inhibition was calculated as described above. The *Z*-factor was calculated as described previously [27]. Cell viability was determined via MTT assays using a commercial kit (Beyotime) according to the manufacturer’s instructions, while the 50% inhibitory concentration (IC_50_), 90% inhibitory concentration (IC_90_), 50% cytotoxic concentration (CC_50_), and selectivity index (SI) values of these drugs were calculated as previously described [28].

### Assessment of anidulafungin inhibition effects on the SFTSV infection process

The inhibitory effect exerted by anidulafungin on the SFTSV infection (MOI = 1) process was assessed using infection assays involving different drug treatment levels (5, 10, and 20 μM) or vehicle at different stages of infection. The inhibitory effect on the entire infection process due to each drug was evaluated using infection assays intended for drug screening, as described above. To determine whether anidulafungin suppresses viral infection by directly interacting with the virus (virion stability), SFTSV was premixed with each dilution of the drug and incubated at 37 °C for 1 h, following which the mixture was added to the cells and maintained at 37 °C for 1 h. Next, the mixture was replaced with a fresh medium, and the cells were maintained as described above. To clarify whether anidulafungin inhibits the entry of SFTSV into cells (virus entry), Vero cells were incubated with a mixture containing both SFTSV and anidulafungin at 37 °C for 1 h, following which the mixture was replaced with a fresh medium. To determine whether anidulafungin inhibits SFTSV replication, the cells were incubated with SFTSV at 37 °C for 1 h. After a medium exchange, cells were maintained in the presence of anidulafungin at 37 °C. In all assays, cells were maintained at 37 °C after the medium exchange and harvested for viral RNA quantification at 24 h p.i.

The effects of anidulafungin on virus binding and internalization were determined using a SFTSV binding assay and an internalization assay, as previously described [25]. Briefly, Vero cells were pretreated with anidulafungin or vehicle at 37 °C for 1 h. The cells were then maintained at 4 °C for 15 min. Subsequently, SFTSV was added, and cells were incubated with a mixture containing virus and anidulafungin or vehicle at 4 °C for 1 h. In order to quantify SFTSV binding to cells, cells were harvested after three washes with pre-cooled phosphate buffer saline (PBS, pH 7.2), and the viral RNA copies were determined by qRT-PCR (see Additional file 1: Supporting information) [29]. To determine the extent of virus internalization by cells, the cells that had been washed thrice with PBS were transferred from 4 to 37 °C, maintained in the fresh medium containing NH_4_Cl (20 mM) for 3 h, and harvested for the purpose of viral RNA copy detection via qRT-PCR.

The effects of anidulafungin on the entry of SFTSV via the endocytosis pathway were evaluated using virus infection assays in combination with inhibitors that blocked clathrin-mediated endocytosis (CPZ, 35 μM), caveola-mediated endocytosis (nystatin, 40 μM), or macropinocytosis (IPA3, 30 μM). Briefly, Vero cells were pretreated with an inhibitor for 2 h followed by incubation with SFTSV (MOI =5) and anidulafungin (12.5 μM) for 1 h at 37 °C. After washing thrice with PBS, the cells were maintained in the fresh medium for 24 h. Next, Western blotting was performed to detect viral protein expression in cells, and inhibition rates were calculated, as described (see Additional file 1: Supporting information) [30].

### Negative staining electronic microscopy (EM) analysis

The cell debris-clarified supernatants (10 mL) were centrifuged to concentrate virions by using the 10,000 MWCO ultrafiltration tube (Millipore, MA, USA). Titers were determined by end-point dilution assays. Then, purified SFTSV virions (4.53 × 10^6^ TCID_50_) were incubated with vehicle or different doses of anidulafungin (20, 50, and 100 μM) at 37 °C for 1h. Then, negative staining of viral particles was performed and visualized as previously described [26].

### Endosomal pH measurement

The endosomal pH in Vero cells incubated with SFTSV during the entry stage was measured according to the protocol as previously described [13, 31]. Briefly, Vero cells were incubated with SFTSV (MOI = 1) at 37 °C for 15, 30, 45, or 60 min, and then, the cells were incubated with 25 μM LysoSensor yellow/blue DND-160 (Invitrogen) to label the acidic organelles at 37 °C for 5 min. After washes, the LysoSensor signals in cells were immediately measured at the excitation/emission wavelengths of both 390/435 (blue) and 390/525 nm (green) at each time point. The green/blue ratio was calculated as previously described [13, 31], and the pH values were determined based on the standard curve between the ratio and pH. The standard curve was established according to a previous study [32]. Briefly, Vero cells were incubated with LysoSensor yellow/blue for 5 min and then treated with MES buffer (115 × 10^−3^ M KCl, 5 × 10^−3^ M NaCl, 25 × 10^−3^ M MES, and 1.2 × 10^−6^ M MgSO_4_) of different pH (4.0–7.0) for 10 min. The standard curve between the green/blue ratio and pH values was fitted based on a Boltzmann Sigmoid model in GraphPad Prism (version 7, GraphPad Software, San Diego, CA, USA).

### Fusion kinetics and syncytium formation assays

The kinetics of fusion triggered by SFTSV with the cell membrane in the presence or absence of anidulafungin was investigated using the method slightly modified according to the previous study [33]. Briefly, the concentrated SFTSV virions (3.57 × 10^8^ TCID_50_/mL) were labeled with R18 at room temperature for 1 h and filtered through a 0.22-μm filter to remove excess dye. The R18-labeled virions (MOI > 100) were incubated with pre-cooled Vero cells (2 × 10^4^/well) in the presence of anidulafungin or vehicle at 4 °C for 1 h to allow virus binding to the cell membrane. Then, cells were washed and harvested. Viral protein expression in cells was analyzed by Western blot or IFAs. Cells were also treated with citrate-phosphate buffer (0.1 M citric acid, 0.2 M sodium dihydrogen orthophosphate, pH 5.0) together with or without anidulafungin for 15 min to initiate membrane fusion. The fusion-induced R18 dequenching was measured at the excitation/emission wavelengths of 560/590 nm using an automatic microplate reader (Biotek Synergy Hi, USA). The fusion reaction was terminated by adding 0.2% Triton X-100 to release the maximal dequencing capacity. The percent of fusion-induced R18 dequenching after the low-pH treatment for 10 min was normalized to the fluorescence measured after adding 0.2% Triton X-100.

Vero cells were infected with SFTSV (MOI = 5), cultured for 45 h at 37 °C, and then pretreated with or without anidulafungin for 3 h, followed by incubation with citrate-phosphate buffer (0.1 M citric acid, 0.2 M sodium dihydrogen orthophosphate, pH 5.0) for 15 min. Cells were then washed and cultured with the fresh medium until 24 h.p.i. To visualize syncytium formation following treatment with anidulafungin or vehicle, the SFTSV-infected cells were immunostained via IFA using in-house prepared anti-SFTSV NP antibody, as previously described [17], while the nuclei were stained with DAPI. Images were captured using an inverted fluorescence microscope. Fusion ability was expressed as the number of cells that formed syncytia over the number of SFTSV-infected cells.

### Confocal microscopy

Vero cells were transfected with plasmids pEGFP-Cla, pEGFP-Rab5, and pEGFP-Rab7 [13], respectively, using Lipofectamine 3000 according to the manufacturer’s instructions (Invitrogen) to label clathrin, EEs, and LEs. At 36 h post-transfection, cells were incubated with SFTSV (MOI = 5) and anidulafungin (12.5 μM) at 37 °C. Culture supernatants were removed at 15, 30, 45, and 60 min post-incubation. Cells were washed thrice with PBS and fixed with 4% paraformaldehyde. The nuclei were stained with DAPI. Fluorescence images were obtained using a laser-scanning confocal microscope (× 60, Nikon A1 MP STORM, Tokyo, Japan).

### SFTSV infection and anidulafungin treatment of A129 mice

A129 mice (type I interferon receptor-deficient) were maintained in an environmentally controlled, specific pathogen-free (SPF) animal facility at the Laboratory Animal Center of Wuhan Institute of Virology, Chinese Academy of Science. Six to 8-week-old male A129 mice (*n* = 6 per group) were intraperitoneally inoculated with serial dilutions (0.01, 0.1, 1, or 10, TCID_50_ per mouse) of the SFTSV strain WCH, in a virus-sustainable supernatant (DMEM containing 2% FBS). Signs of illness onset and body weight were monitored for 14 days, following which the survival rates and median lethal dose (LD_50_) were calculated. Subsequently, male A129 mice (*n* = 6 per group) were inoculated with SFTSV (1 or 10 LD_50_ per mouse) or an equivalent volume of virus culture supernatant as a mock control. At 1 h post-SFTSV challenge, the mice were intraperitoneally administered a tolerable dose of 10 mg/kg anidulafungin or vehicle (a mixture of 5% DMSO, 40% PEG300, and 5% Tween 80 in ddH_2_O, filtered through a 0.22-μm filter) per mouse, every day for 14 days. Mice were monitored for signs of disease, and the body weight of each mouse was recorded for 14 days. On days 0, 3, 7, and 14 following SFTSV inoculation, blood samples were collected for routine blood tests and serum separation. Blood biochemistry tests were performed on approximately 100 μL serum from each mouse, to analyze the levels of AST, ALT, and BUN using a Hitachi 700-210 analyzer (Hitachi medical, Gyoonggi-do, South Korea). Tissues were collected from survived mice on days 3, 7, and 14 after SFTSV infection and were fixed in 4% paraformaldehyde for 24 h. Hematoxylin and eosin (H&E) staining and immunohistochemical staining were performed as previously described [17]. SFTSV RNA loads were determined by qRT-PCR. To investigate the effects of anidulafungin given to mice at a later time point, the A129 mice (*n* = 6 per group) were inoculated with SFTSV intraperitoneally (LD_50_) and administrated with anidulafungin after 1, 2, or 3 days. The mice were monitored within 14 days to inspect their survival and change in body weights. Survived mice were euthanized by the end of experiments. The experiments were performed in accordance with the NC3Rs ARRIVE guidelines (see Additional file 2) [34, 35]

### *Antiviral activity of anidulafungin against the entry process of* SARS-CoV-2, GTV, HRTV, ZIKV, CCHFV, and HSV-1 infection

Vero cells were incubated at 37 °C for 1 h with a medium containing both the virus (MOI = 1) and the drug in serial dilutions of 25, 12.5, 6.25, 3.125, 1.5625, and 0.78125 μM, respectively. The supernatants were then replaced with fresh medium, and cells were further cultured for 24 h. SARS-CoV-2, GTV, HRTV, ZIKV, CCHFV, and HSV-1 infections in cells treated with anidulafungin or vehicle were identified via IFAs using in-house prepared rabbit polyclonal antibodies including anti-SARS-CoV-2 NP, anti-SFTSV NP which shows serological cross-reactions with GTV NP [17], anti-HRTV NP, and anti-CCHFV NP, or mouse monoclonal antibodies including 4G2 specific to the flavivirus group antigen and m27f targeting HSV glycoprotein D (gD). Fluorescence images were captured, and for each test, infection rates were calculated as described above. Each test was performed in triplicate.

### Data analyses

The statistical significance of differences between the two groups was determined using Student’s *t*-test. Statistical analyses were performed by GraphPad Prism (version 7, GraphPad Software). *P* values < 0.05 were considered statistically significant.

## Results

### Screening of FDA-approved drugs identified compounds with anti-SFTSV activity

**Fig. 1:**
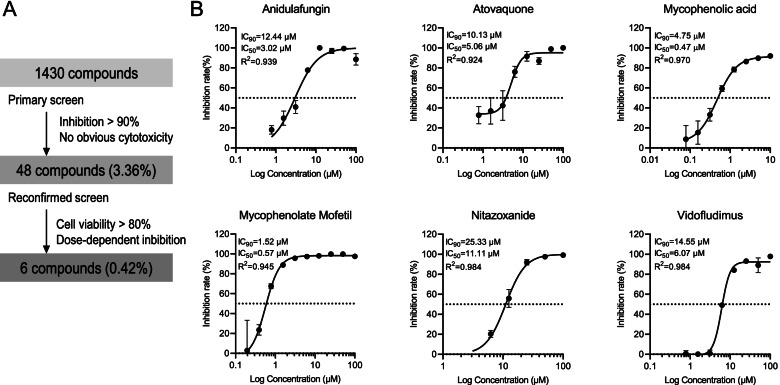
Cell-based high-throughput screening of FDA-approved drug library identified anidulafungin as an inhibitor of SFTSV infection. **A** The flowchart of high-throughput screening protocol used to identify inhibitors against SFTSV infection. **B** Dose-dependent inhibitory effects of six identified compounds revealed via cell-based high-throughput screening of Vero cells

We screened a library of 1430 FDA-approved drugs for anti-SFTSV activity (Fig. 1A). The primary screen identified 48 compounds that significantly inhibited virus infection (inhibition rate > 90%) at a concentration of 100 μM, without causing obvious cytotoxicity. A *Z*-factor of 0.80 indicated that primary screening was robust. The second screen confirmed that 6 of the 48 compounds exerted anti-SFTSV activity in a dose-dependent manner (all *R*^2^ > 0.900; Fig. 1B) without causing cytotoxicity (cell viability > 80%; Fig. 1A). The calculation of SI values of these 6 drugs was based on IC_50_ and CC_50_ in Vero cells, which showed that all six drugs exhibited strong antiviral activity with low cytotoxicity (SI values > 10; see Additional file 3: Table S1).

### Anidulafungin and its analogs inhibit SFTSV infection by interfering with the virus entry process

**Fig. 2:**
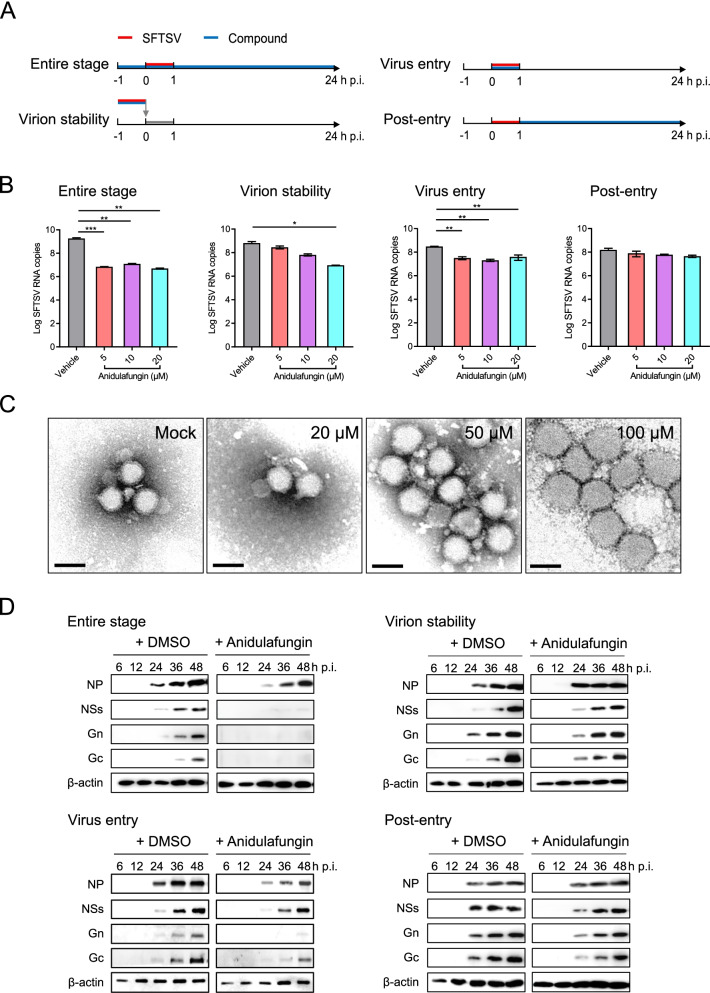
The inhibitory effect of anidulafungin on different stages of SFTSV infection was evaluated. **A** Flowcharts based on SFTSV infection assays identified antiviral activity of selected compounds that interfere with the entire stage, virion stability, virus entry, or post-entry stage of SFTSV infection in Vero cells. **B** The number of intracellular SFTSV RNA copies in Vero cells produced with or without anidulafungin treatment during different stages of the infection assay as measured by qRT-PCR. **C** Negative-staining EM analyses to visualize SFTSV virions incubated with anidulafungin (20, 50, and 100μM) or vehicle. Bars, 100 nm. **D** SFTSV protein expression in Vero cells with or without anidulafungin at the different stages was analyzed using Western blot. The significant difference was determined using Student’s *t*-test. *P < 0.05; **P < 0.01; ***P < 0.001; ****P < 0.0001

The mechanism that enabled the above described 6 drugs to inhibit SFTSV infection was investigated, in terms of ability to disrupt virion stability, virus entry, and post-entry process (Fig. 2A). Five of the six drugs inhibited SFTSV infection when added to the culture medium after SFTSV incubation (post-entry stage) (unpublished data), whereas anidulafungin was observed to exert inhibitory effect on the virus entry process (virus entry) as similar to the entire infection process (entire stage), as evidenced by the reduced intracellular viral RNA copies by over 10 times that of the control (Fig. 2B). A decrease in the number of intracellular viral RNA copies was also noted when the virus was pre-incubated with a high dose of anidulafungin before being added to the cell culture medium (virion stability) (Fig. 2B). Such inhibition by anidulafungin seemed to be dose-dependent, however was only significantly effective at a high dose compared to that for virus entry (*P* = 0.0347) (Fig. 2B). EM analyses showed that the diameter of SFTSV virions with incubation of anidulafungin at a low concentration (20μM) was approximately 80 nm, which is as similar as that of the virions without anidulafungin treatment. However, enlarged sizes of SFTSV virions were observed when treated with increasing doses of anidulafungin (50 μM and 100 μM), resulting in the diameters of 90–100 nm and 110–120 nm, respectively (Fig. 2C). These suggested that high dose of anidulafungin may disrupt SFTSV virions, thus impair virus stability and suppress SFTSV infection. Western blot assay and quantitative analyses of protein expression further indicated that treatment with anidulafungin throughout the entire process or during the virus entry stage delayed and also decreased SFTSV protein expression in Vero cells (Fig. 2D, left panels, Additional file 4: Fig. S1A). This impairment of viral protein expression by anidulafungin was not as significant as on virion stability or during the post-entry stage (Fig. 2D, right panels, Additional file 4: Fig. S1B). In addition, a similar inhibitory effect of anidulafungin was observed in Huh7 and HEK 293 cells, the human-deriving cell lines permissive for SFTSV infection which are commonly used to study the mechanisms of SFTSV infection and pathogenesis (Additional file 4: Fig. S2A). These results demonstrated that anidulafungin strongly inhibited SFTSV infection by interfering with virus entry.

Since anidulafungin is a member of the echinocandin family, we further investigated the effects of micafungin and caspofungin, two analogs of anidulafungin, on SFTSV infection during the entry process. Micafungin exerted an inhibitory effect on SFTSV infection, which was less effective than that exerted by anidulafungin, while caspofungin exerted a limited effect on SFTSV infection (Additional file 4: Fig. S2B).

### Anidulafungin interferes with SFTSV entry by suppressing the process of virus internalization

**Fig. 3:**
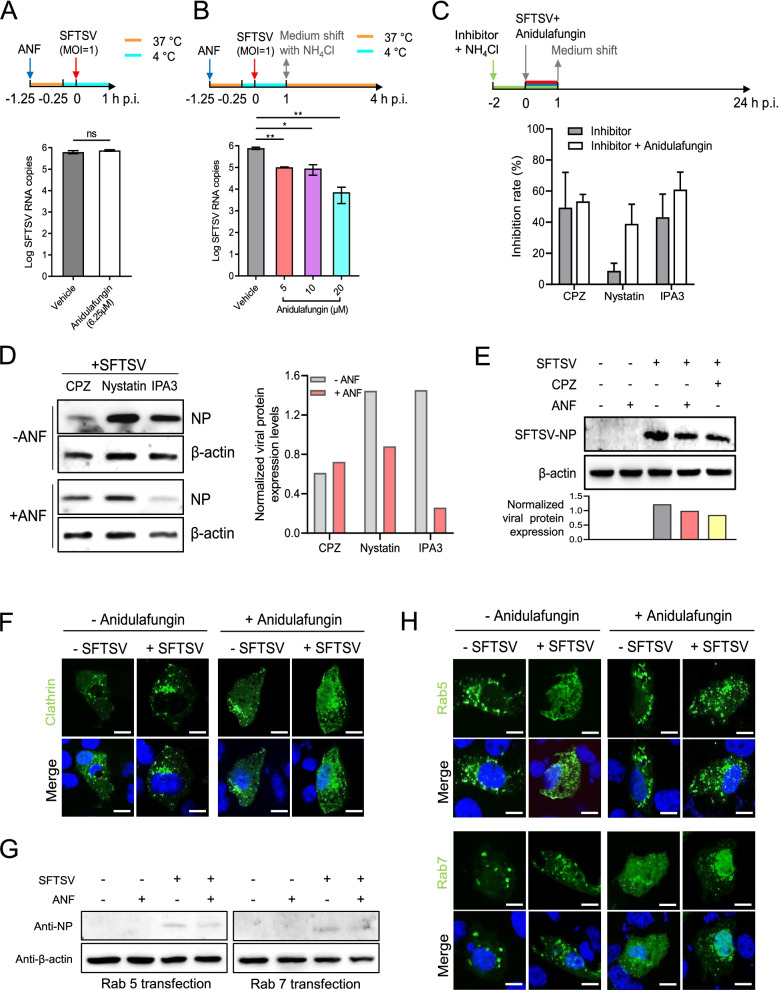
Anidulafungin inhibits SFTSV infection by interfering with virus internalization. **A** The binding assay under a temperature shift was performed to determine the inhibitory effect of anidulafungin on SFTSV binding to Vero cells. **B** The inhibitory effect of anidulafungin on SFTSV internalization into Vero cells was characterized based on the internalization assay under a temperature shift with the treatment of NH_4_Cl. All qRT-PCR tests were performed in triplicates for each sample. **C** The inhibitory effects of anidulafungin on SFTSV internalization into Vero cells were investigated in the presence of inhibitors suppressing the clathrin-mediated endocytosis (CPZ), caveola-mediated endocytosis (nystatin), or macropinocytosis (IPA3). IFAs were performed to characterize the efficiencies of SFTSV infection with the treatment of inhibitors together with or without anidulafungin during the entry stage. Inhibition (%) on SFTSV was calculated by the number of non-infected cells over the number of total nuclei. Each test was performed in triplicate. **D** SFTSV protein expression levels in Vero cells infected with SFTSV were characterized by Western blot with the treatment of inhibitors together with or without anidulafungin (left). The detection of β-actin expression was set as the inner control. Viral protein expression levels were quantified by ImageJ and normalized to the levels of β-actin (right). **E** SFTSV NP expression levels in Vero cells infected with or without SFTSV, treated with or without anidulafungin, or CPZ (upper). SFTSV NP expression levels were quantified by ImageJ and normalized to the levels of β-actin (bottom). **F** Fluorescence images of Vero cells overexpressing clathrin were taken to determine the intracellular distributions in cells with or without treatment of anidulafungin. Cells were transfected with clathrin expressing plasmid. At 24 h post-transfection, these cells were incubated with SFTSV (MOI = 1) together with or without treatment of anidulafungin at 37 °C. Images were taken at 30 min post-virus incubation. Bars, 5 μm. **G** SFTSV NP was detected by Western blot in Vero cells transfected with the Rab5 or Rab7 expression plasmids. Cells were transfected with Rab5 or Rab7 expressing plasmids. At 24 h post-transfection, these cells were incubated with SFTSV (MOI = 1) together with or without treatment of anidulafungin at 37 °C for 1 h. Then, cells were harvested, and Western blot was performed to detect SFTSV NP expression. **H** Fluorescence images of Vero cells overexpressing Rab5 or Rab7 were taken at 30 min post-SFTSV incubation to determine their intracellular distributions in cells with or without treatment of anidulafungin as described in **G**. Bars, 5 μm

The mechanism underlying the inhibition of SFTSV entry by anidulafungin was further characterized. The binding assay was performed and viral RNA binding to cells was determined by qRT-PCR. The results revealed that the efficacy of virus binding to cells in the presence of anidulafungin did not have a significant difference from that of cells incubated with vehicle (*P* = 0.1024) (Fig. 3A), suggesting that anidulafungin did not affect the binding of SFTSV to cellular receptors. In order to determine the efficacy of virus internalization, cells were incubated with viruses at 4 °C for 1 h, following which the cell culture supernatant was replaced with a fresh medium containing ammonium chloride (NH_4_Cl) to prevent membrane fusion, and the cells maintained at 37 °C for 3 h. The qRT-PCR analysis showed that anidulafungin had significantly decreased the number of intracellular viral RNA copies (5 μM: *P* = 0.0090; 10 μM: *P* = 0.0108; 20 μM: *P* = 0.0070) (Fig. 3B). This suggested that anidulafungin interfered with the process of SFTSV internalization.

SFTSV internalization by host cells is initiated by recruiting clathrin to the plasma membrane to form clathrin-coated pits, which further pinch off to form discrete vesicles [13]. Therefore, we speculated that anidulafungin may inhibit SFTSV internalization by interfering with the clathrin-mediated endocytosis pathway. While fusion with the plasma membrane was inhibited by NH_4_Cl, pretreatment of cells with CPZ, an inhibitor of clathrin-mediated endocytosis, and IPA3, an inhibitor of macropinocytosis, inhibited SFTSV infection, resulting in the mean inhibition rates of 46.06% and 43.22%, respectively. Pre-treatment with nystatin, an inhibitor of caveola-mediated endocytosis, resulted in the mean inhibition rates of 8.67%. Moreover, although anidulafungin did not further promote the inhibitory effects of CPZ on SFTSV infection, it did increase the inhibition rate together with the treatment of nystatin or IPA3 (Fig. 3C). This enhancement of inhibitory effects was also confirmed by Western blot analysis (Fig. 3D, left panel). The expression levels of SFTSV nucleoprotein (NP) decreased further following treatment by anidulafungin together with nystatin or IPA3 (Fig. 3D, right panel). The efficiency with which anidulafungin treatment during the entry stage decreased SFTSV NP expression was similar to that of the CPZ treatment (Fig. 3E), whereas a pre-treatment of anidulafungin together with CPZ did not diminish the protein expression (Fig. 3D). These results confirmed that anidulafungin inhibits SFTSV internalization by targeting the clathrin-mediated endocytosis pathway as efficiently as CPZ but does not significantly enhance the inhibitory effect of CPZ. SFTSV infection promoted the recruitment of clathrin and the formation of clathrin-coated vesicles (CCVs) [13], as evidenced by clathrin puncta and clusters that were observed close to the cell membrane (Fig. 3F, left panels). Anidulafungin treatment exerted a limited effect on the recruitment of clathrin, as indicated by the fact that clathrin puncta located close to the plasma membrane in cells incubated with SFTSV were still increasing (Fig. 3F, right panels).

The transport of SFTSV particles into the cytosol involves sequential delivery from CCVs to Rab5-positive early endosomes (Rab5+ EEs) and then to Rab7-positive late endosomes (Rab7+ LEs) [13]. Subsequently, we characterized the intracellular distribution of Rab5 and Rab7 with or without anidulafungin, as these are essential for endosome formation. Although in a limited protein expression level, SFTSV infection in the Rab5 and Rab7 plasmid-transfected Vero cells was confirmed by Western blot at 60 min post-incubation (Fig. 3G). Rab5 was expressed in the form of puncta scattered in the cytosol (Fig. 3H; upper panels), but there was no significant difference between Rab5 distribution in the cells of the four treatments: −anidulafungin/-SFTSV, −anidulafungin/+SFTSV, +anidulafungin/−SFTSV, and +anidulafungin/+SFTSV. More vascular structures formed by Rab7 were found in the cytosol at a similar time point following SFTSV incubation. Following anidulafungin treatment, Rab7 was mostly dispersed in cells with a few vascular structures in the cytosol. A similar impact to Rab7 in cells with anidulafungin treatment after SFTSV infection was also noted (Fig. 3H, bottom panels). These results were further confirmed by continuous inspection of the intracellular distribution of Rab5 and Rab7 in the presence of anidulafungin at 15, 30, 45, and 60 min after SFTSV incubation (Additional file 4: Fig. S3). Rab5 puncta and clusters were generally scattered in the cytosol regardless of whether cells were treated with anidulafungin or not (Additional file 4: Fig. S3A). Although vascular structures were found in cells, a proportion of Rab7 was dispersed in the nuclei of an increasing number of cells after 30 min of incubation with SFTSV (Additional file 4: Fig. S3B). During the SFTSV entry stage within 60 min post-incubation, the average pH value in cells treated with anidulafungin was 5.87 ± 0.08, which is as comparable as that in cells treated with DMSO (5.89 ± 0.04). At each time point of 15, 30, 45, and 60 min post-infection, the pH values did not exhibit a significant difference between cells treated with DMSO and anidulafungin (Additional file 4: Fig. S4). These results suggest that anidulafungin may interfere with SFTSV internalization by impairing the maturation of Rab7+ LEs; however, it did not exhibit obvious impact on endosomal acidification.

**Fig. 4:**
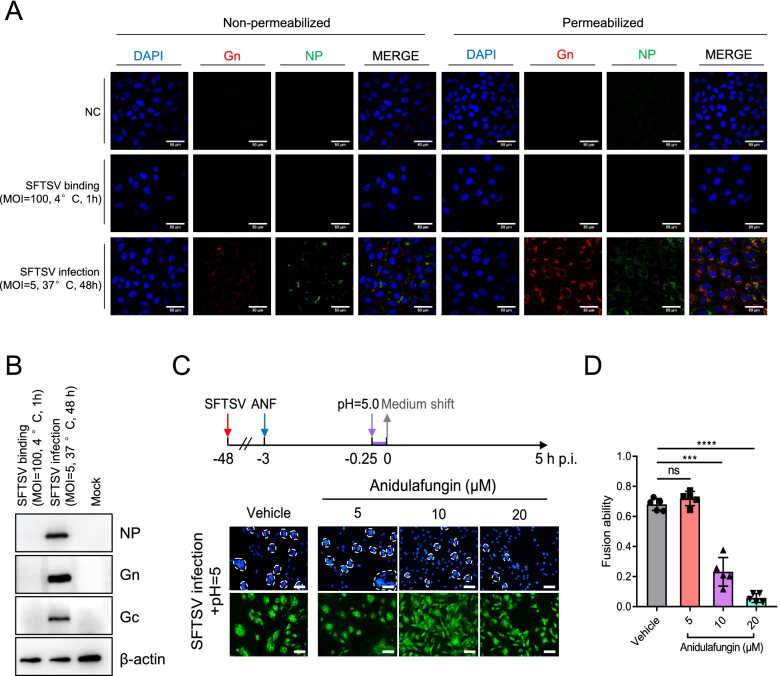
The inhibitory effect of anidulafungin on SFTSV fusion with the cell membrane. **A** SFTSV NP and Gn expression both on the cell surface and in cells were detected by IFAs. Vero cells were treated with anidulafungin (20 μM for 1 h and then incubated with SFTSV (MOI = 100) at 4 °C for 1 h, or infected with SFTSV (MOI = 25)) and maintained at 37 °C for 48 h. After washes, IFAs were performed to detect viral protein on the cell surface without treatment of 0.2% Triton X-100 (non-permeabilized) and in cells with treatment of 0.2% Triton X-100 (Permeabilized). Bars, 50 μm. **B** Western blot was performed to analyze SFTSV NP, Gn, and Gc expression in Vero cells incubated with SFTSV (MOI = 100) at 4 °C for 1 h or cells infected with SFTSV (MOI = 5) at 37 °C for 48 h. Mock, healthy cells. **C** Syncytia formation assays were performed to investigate the inhibitory effects of anidulafungin on SFTSV fusion ability triggered by acidic conditional. The infected Vero cells were indicated by immunostaining SFTSV NP expression, and cells were indicated by staining nuclei. Bars, 200 μm. **D** The fusion ability of SFTSV in the presence or absence of anidulafungin was expressed by the number of cells that form syncytium over the number of SFTSV-infected cells from three images of each test. h p.i., hours post-incubation. The significant difference was determined using Student’s *t*-test. *P < 0.05; **P< 0.01; ***P < 0.001; ****P < 0.0001

The final fusion events that enable SFTSV to release RNA into the cytosol occur within the LEs and are triggered by liminal acidic pH [13]. We tried to quantify the effect of anidulafungin on fusion kinetics by measuring the fluorescence of fusion-induced R18 dequenching but failed to detect increased signals of R18 dequenching induced by R18-labeled SFTSV (MOI > 100) fusion with cell membrane after 1h incubation at 4 °C (data not shown). SFTSV protein expression was identified by IFAs neither on the cell surface nor in cells after SFTSV binding (MOI = 100), while the expression was confirmed both on the cell surface and in cells with SFTSV infection (MOI = 5) at 48 h p.i. (Fig. 4A). Similar results were also identified by Western blot, which showed SFTSV NP, Gn, and Gc expression in the infected cells but not in cells with SFTSV binding (Fig. 4B). These explained that syncytia formation, triggered by a low pH shift in the medium, was observed from cells with SFTSV infection (Fig. 4C, vehicle), however, not found from cells after SFTSV binding (data not shown). Moreover, the number and size of syncytia decreased as the anidulafungin dosage applied to the SFTSV-infected cells was increased (Fig. 4C). The decrease in fusion ability caused by anidulafungin treatment was demonstrated by the decreasing ratios of the number of cells forming syncytia over the number of cells with SFTSV infection (5 μM: *P* = 0.1934; 10 μM: *P* = 0.0001; 20 μM: *P* < 0.0001) (Fig. 4D). These results revealed that sufficient SFTSV glycoprotein expression on the cell surface is associated with membrane fusion and that anidulafungin could inhibit virus entry by suppressing viral fusion with the membrane.

### Anidulafungin protects mice from SFTSV infection

**Fig. 5:**
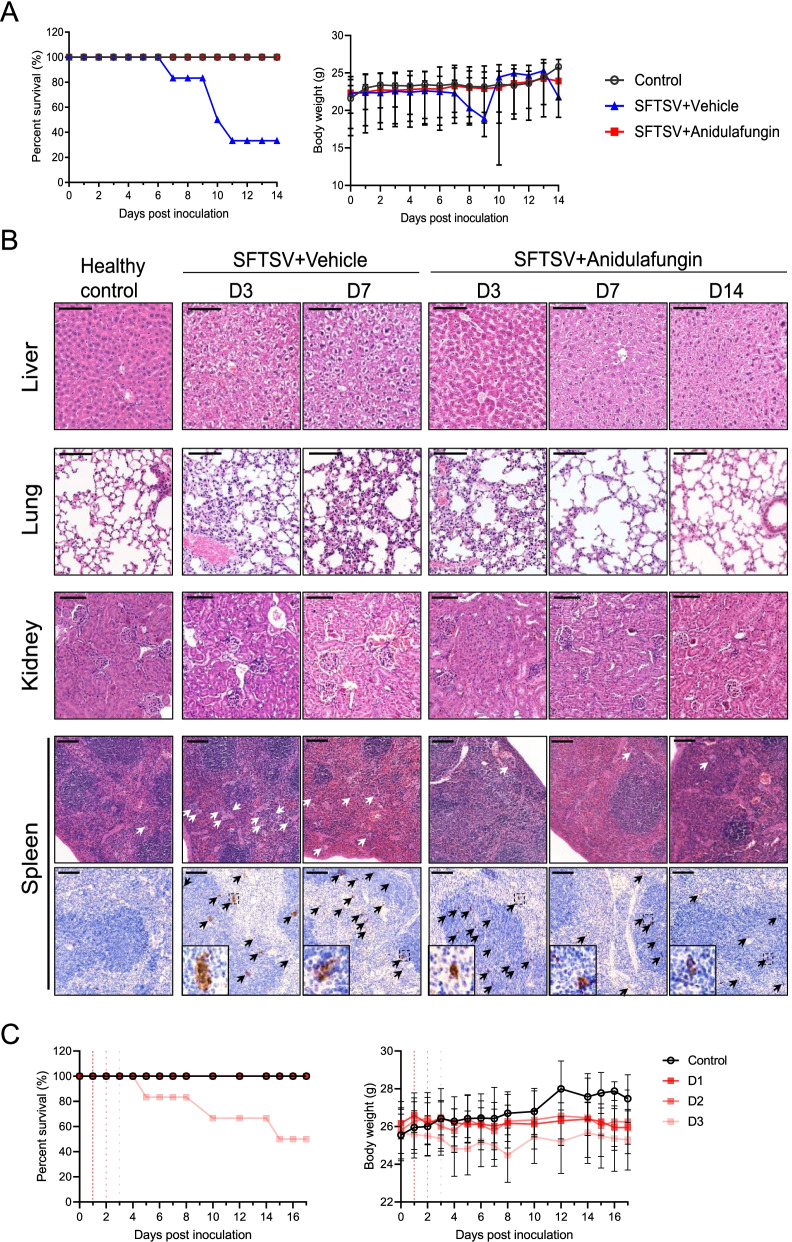
The animal experiments with the treatment of anidulafungin. **A** The percent of survival and body weight changes (median and range) were monitored for 14 days among the A129 mice challenged by SFTSV doses of 1 LD_50_ and with the treatment of anidulafungin or vehicle. **B** The H&E assays and immunohistochemistry staining revealed the pathological lesions in the liver, lung, kidney, and spleen of A129 mice treated with anidulafungin or vehicle and SFTSV infection in the kidney, respectively. Tissues were harvested on days 3 and 7 from mice of the vehicle group and the anidulafungin group after SFTSV infection. On day 14, tissues were collected from the survived mice of the anidulafungin group. Megakaryocytes in the spleen were indicated by white arrows, and the cell foci positive for SFTSV antigen, black arrows. Bars, 100 μm. **C** The percent of survival and body weight changes (median and range) of A129 mice were monitored within 14 days, which were challenged by SFTSV doses of 1 LD_50_ and were also given with anidulafungin on day 1, 2, or 3 after challenge

We investigated whether administering anidulafungin protects mice from SFTSV infection. Mice in the group, which were inoculated with SFTSV at a dose of 1 LD_50_ and administered vehicle, showed first illness onset on day 7. Only two of the six mice (33.33%) survived by day 14. Mice inoculated with a similar dose of viruses and administered anidulafungin did not show typical clinical signs of illness onset, and all mice in the group (100%) survived. The body weight of mice administered vehicle decreased, whereas that of mice administered anidulafungin was maintained (Fig. 5A). The results of routine blood tests showed that white blood cell (WBC) counts in mice infected with SFTSV had not decreased by day 7, probably because this was tested from the surviving mice, whereas the WBC counts in mice receiving anidulafungin therapy decreased on days 3 and 7 and recovered almost to the control level by day 14. Mice administered vehicle or anidulafugin had decreased platelet (PLT) counts on days 3 and 7. The PLT counts of mice receiving anidulafungin increased on day 14, despite the levels being less than those before SFTSV infection (Additional file 3: Table S2). In addition, the pathological changes induced by SFTSV infection in the organs were evaluated by H&E staining (Fig. 5B). Ballooning degeneration of hepatocytes was observed in the liver on day 3 after SFTSV infection in mice treated by vehicle and was worse by day 7. A similar lesion was also noted in the liver of mice treated with anidulafungin on day 3 and was significantly attenuated on days 7 and 14. SFTSV infection resulted in lesions in the lung including hemorrhaging in alveolar spaces and extensive infiltration of inflammatory cells, which was also found in the lung of mice treated with anidulafungin on day 3. The treatment of anidulafungin significantly ameliorated the histopathological damage in the lung on days 7 and 14. The mice treated with vehicle had renal tubulointerstitial hemorrhaging and edemas of renal tubular epithelial cells in the kidneys, while these lesions were slight in mice treated by anidulafungin. SFTSV infection induced increasing numbers of megakaryocytes in the red pulp of the spleen, while this increase was not significant for mice with anidulafungin administration. Moreover, immunohistochemical staining for SFTSV antigen expression identified cell foci with SFTSV infection in the spleen. Although viral antigen expression were still detected in the spleen on day 14, the reduced sizes and decreased numbers of cell foci might suggest that anidulafungin therapy could suppress SFTSV spreading in the tissues (Fig. 5B). However, we tried but failed to detect SFTSV RNA in the spleens from these mice by qRT-PCR, probably due to the limited infection of SFTSV resulting in the undetectable levels of viral RNA comparing with the high abundance of mouse tissue RNA. Nevertheless, anidulafungin also showed a protective effect on the mice infected with SFTSV at a dose of 10 LD_50_, resulting in a survival rate of 66.7%, while only one mouse survived in the vehicle group (Additional file 4: Fig. S5). Moreover, when mice infected with SFTSV (1 LD_50_) were administrated with anidulafungin 1 or 2 days post-infection, all of them survived, whereas those administrated with anidulafungin 3 days after SFTSV infection resulted in the survival rate of 50.0% (Fig. 5C, left). The mice treated with anidulafungin 3 days after infection showed a slight decrease in body weight, while the body weight of other groups was maintained throughout the 14 days (Fig. 5C, right). All these results showed that anidulafungin could reduce lethality and improve the outcomes of SFTSV infection in mice.

### Anidulafungin may be a broad-spectrum inhibitor that suppresses infection of a wide range of viruses

**Fig. 6:**
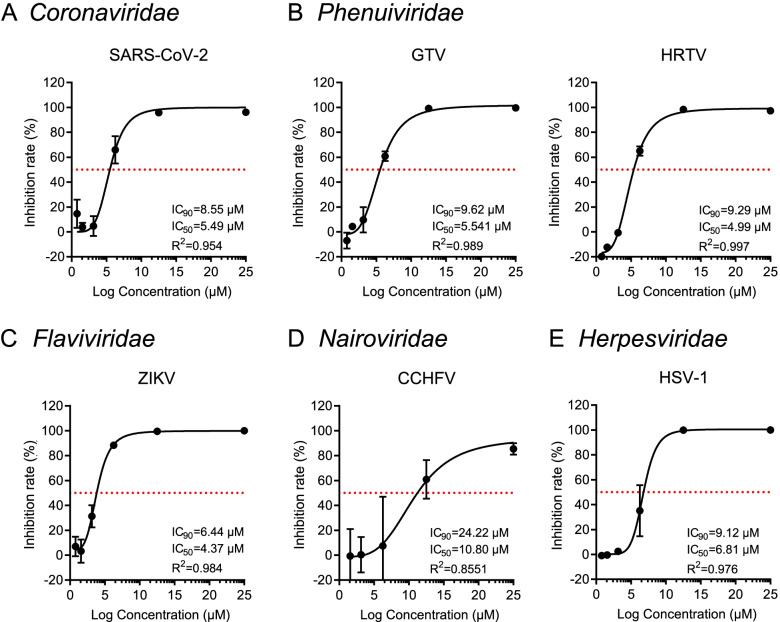
The broad-spectrum antiviral activity of anidulafungin. The inhibitory effects of anidulafungin on the entry stage of infection in Vero cells by **A** SARS-CoV-2 belonging to the family *Coronaviridae*, **B** GTV and HRTV to *Phenuiviridae*, **C** ZIKV to *Flaviviridae*, **D** CCHFV to *Nairoviridae*, and **E** HSV-1 to *Herpesviridae* were measured in a dose-dependent manner

We speculated that anidulafungin may also inhibit other viruses. Therefore, the inhibitory effects exerted by anidulafungin on the virus entry stage were investigated using viruses belonging to different viral families. Infections of SARS-CoV-2 (*Coronaviridae*), the SFTSV-related viruses (GTV and HRTV (*Phenuiviridae*)), ZIKV (*Flaviviridae*), CCHFV (*Nairoviridae*), and HSV-1 (*Herpesviridae*) were effectively inhibited by anidulafungin treatment in a dose-dependent manner (Fig. 6). The IC_50_ values for anidulafungin-based inhibition of these viruses were calculated as follows: ZIKV (4.37 μM) < HRTV (4.99 μM) < SARS-CoV-2 (5.49 μM) < GTV (5.54 μM) < HSV-1 (6.81 μM) < CCHFV (10.80 μM), while the order of IC_90_ values for these viruses were ZIKV (6.44 μM) < SARS-CoV-2 (8.55 μM) < HSV-1 (9.12 μM) < HRTV (9.29 μM) < GTV (9.62 μM) < CCHFV (24.22 μM) (Fig. 6; all *R*^2^ > 0.850). Despite the SI values to the tested viruses < 10 (Additional file 3: Table S3), the results still suggested that anidulafungin exerts broad-spectrum inhibitory effects that suppress virus infection by interfering with virus entry into host cells.

## Discussion

Much effort has been made to screen and identify candidate antiviral drugs and to evaluate their inhibitory activity against SFTSV in vitro as well as in vivo [36]. The broad-spectrum antiviral drug, ribavirin, was expected to be an option for the clinical therapy of SFTS. However, this drug was ineffective in improving clinical outcomes, and an increase in common adverse events was observed in patients receiving ribavirin therapy [11]. Favipiravir is a RNA chain terminator that introduces a mutagen lethal to RNA viruses in vitro [37], which has been shown to inhibit SFTSV replication both in vitro and in animal models [38]. Recently, the clinical effect of favipiravir in treating SFTS has been assessed in a single-blinded, randomized controlled trial, which showed that the administration of favipiravir can reduce fatality in SFTS patients [39]. Screening of the FDA-approved drug library revealed that the calcium channel blockers, benidipine hydrochloride and nifedipine, exerted inhibitory effects on SFTSV infection, and a retrospective investigation revealed that administering nifedipine improved clinical outcomes and reduced the fatality rate of SFTS [25]. Hexachlorophene, an antibacterial disinfectant also from this drug library, was found to inhibit SFTSV infection, possibly by binding to the deep pocket between domains I and III of the SFTSV Gc glycoprotein and interfering with the viral fusion process [40]. In this study, we screened the FDA-approved drug library containing 1430 compounds and identified six drugs that inhibited SFTSV infection with SI values > 10. Unlike the other five drugs that inhibit SFTSV infection during the post-entry phases (unpublished data), anidulafungin strongly inhibited the SFTSV entry process itself and also affected virion stability at a high dosage in vitro. Moreover, administering anidulafungin improved the outcomes of SFTSV-infected mice and increased their survival rates. These results demonstrated that anidulafungin exerts antiviral effects on SFTSV both in vivo and in vitro.

Endocytosis is a common mechanism used by most viruses to enter host cells. A majority of viruses depend on endocytic uptake, vesicular transport, and delivery to endosomes or other organelles, while some viruses are able to penetrate into the cytosol directly through the plasma membrane [41]. SFTSV internalization is a multistep process involving dynamic virus-host interactions initiated by the clathrin-mediated endocytosis pathway. Clathrin is recruited to the plasma membrane to form clathrin-mediated pits, which then pitch off from the membrane to form clathrin-coated discrete vesicles. Virions in the vesicles are delivered to Rab5+ EEs and then to Rab7+ LEs. Finally, fusion events are triggered by an acidic environment (~ pH 5.6) within LEs [13]. Our study indicated that anidulafungin treatment did not reduce virus binding but significantly reduced SFTSV internalization. Furthermore, the use of inhibitors of caveola-mediated endocytosis or macropinocytosis together with anidulafungin enhanced the inhibition of SFTSV internalization and decreased viral protein expression. However, such enhancement was not observed when an inhibitor of clathrin-mediated endocytosis was used together with anidulafungin. These results confirmed that anidulafungin inhibited SFTSV infection by interfering with clathrin-mediated endocytosis. We characterized the intracellular distribution of clathrin, Rab5, and Rab7, which are essential components for the formation of CCVs and the EE and LE trafficking and maturation in healthy and SFTSV-infected cells, and found that anidulafungin did not significantly affect the intracellular distribution of clathrin puncta and Rab5 clusters. However, the formation of Rab7 vascular structures was impaired by anidulafungin, whereas endosomal acidification was not affected. Furthermore, fusion activity induced by SFTSV was significantly reduced following anidulafungin treatment. These results demonstrated that anidulafungin inhibits SFTSV internalization by interfering with the formation or maturation of Rab7+ LEs and subsequent fusion with the membrane, thus decreasing the efficiency of SFTSV intracellular transport and viral RNA release into the cytosol, which, in any case, is unlikely to be associated with the antifungal functioning to inhibit 1,3-β-d glucan synthesis [42].

**Fig. 7:**
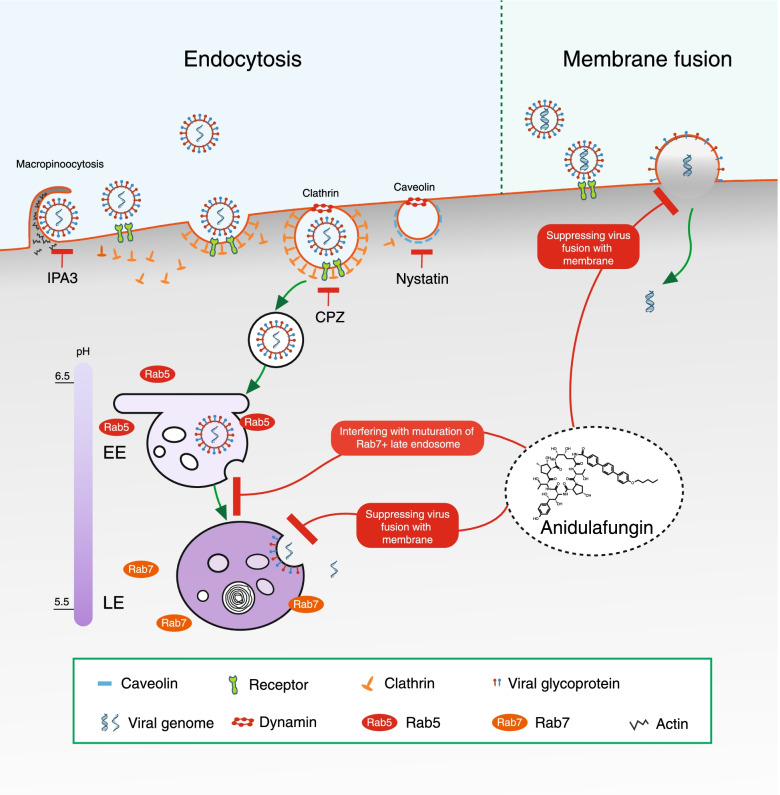
The proposed model for the anidulafungin inhibition on virus internalization via clathrin-mediated endocytosis and membrane fusion

The mechanisms underlying the anidulafungin-induced inhibition of virus entry via clathrin-mediated endocytosis are summarized and illustrated, in comparison with the inhibitors (CPZ, nystatin, and IPA3) to suppress virus entry via clathrin recruitment, caveolin formation, or macropinocytosis (Fig. 7). Our results indicate that anidulafungin may be an effective antiviral drug against viruses similar to SFTSV which utilize endocytosis-based strategies to enter cells. Viruses belonging to *Phenuiviridae* [43] and *Flaviviridae* [44] generally enter cells via clathrin-mediated endocytosis, where membrane fusion occurs within LEs. Although the entry mechanism of nairoviruses has not been well clarified, a previous study showed that CCHFV (the representative viral species of the genus *Orthonairovirus* in the family *Nairoviridae*) enters cells via the clathrin-mediated endocytosis pathway, where membrane fusion is triggered by a moderately acid pH within EEs [45]. As expected, we found that anidulafungin effectively inhibited the entry process of SFTSV-related GTV and HRTV (*Phenuiviridae*), ZIKV (*Flaviviridae*), and CCHFV (*Nairoviridae*). Furthermore, anidulafungin exerted inhibitory effects on the entry process of other viruses, including HSV-1 and SARS-CoV-2, which mainly enter cells via plasma membrane fusion (Fig. 7). HSV-1 is a double-stranded DNA virus belonging to the *Herpesviridae* family, which commonly enters host cells via a direct membrane fusion mechanism triggered by four viral glycoproteins, gD, gH/gL, and gB, binding to specific receptors. HSV entry via a low pH-dependent, atypical endocytic pathway has also been described, but the initial steps remain unclear [46]. SARS-CoV-2 enters cells through plasma membrane fusion or endocytosis, which is dependent on receptor binding and proteolytic activation of spike proteins and affected by cellular attachment cofactors [47, 48]. Therefore, we speculate that anidulafungin treatment is more likely to inhibit HSV-1 and SARS-CoV-2 internalization by interfering with virus fusion with the plasma membrane. This echoes with the recent study that anidulafungin inhibits SARS-CoV-2 spike-induced syncytia formation by targeting ACE2-spike protein interaction [49]. All data suggest that anidulafungin exerts broad-spectrum antiviral effects by suppressing viral entry into host cells.

Synthetic small antifungals could be classified into four major classes, including polyenes, flucytosine, echinocandins, and azoles, according to their differential functional targets and mechanisms [50]. Except for flucytosine which interferes with fungal nucleic acid synthesis, polyenes and azoles show antifungal effects resulting in compromised fungal membranes. Polyenes bind to sterol components and form pores on the membrane, whereas azoles inhibit fungal sterol biosynthesis [50]. Unlike other antifungals, echinocandins exhibit effective antifungal activity by targeting 1,3-β-D glucan synthase. As a result, the synthesis of an essential component of the fungal cell wall, 1,3-β-D glucan, is suppressed, and the fungal cell wall formation is disrupted [42]. Although a cell wall is not in mammalian cells, anti-viral activity in vitro by the members of the echinocandin family, including micafungin, caspofungin, and anidulafungin, has been reported by a few recent studies [51–53]. Micafungin was the most studied echinocandin for exerting effective antiviral activity against different viruses. So far, the mechanisms underlying its effects appear to involve multiple processes but still remain obscure. Micafungin suppresses enterovirus 71 infection by affecting intracellular events involving 2C and 3A proteins, IRES-dependent translation, and polyprotein processing [51]; inhibits chikungunya virus infection by limiting viral replication, release, and cell-to-cell transmission [52]; and reduces the infection efficiencies of dengue virus (DENV) by disrupting virus binding, entry, and stability [53]. In addition, the antiviral activities of echinocandins, leading to the inhibition of DENV infection, were also characterized according to the order of IC_50_ values as follows: anidulafungin (3.24 μM) < micafungin (10.23 μM) < caspofungin (20.78 μM) [53]. This suggested that anidulafungin exhibited better antiviral activity. This result was similar to that of our findings that anidulafungin inhibited SFTSV infection more effectively than either micafungin or caspofungin. Furthermore, the antiviral activity of echinocandins affected different DENV types as well as chikungunya virus strains [52, 53], which was also true for anidulafungin to suppress the entry of SFTSV strain HBMC5 as efficiently as the strain WCH (unpublished data). Furthermore, molecular docking analyses and in vitro experiments identified micafungin and the natural echinocandin product, pneumocandin B_0_, as potential inhibitors of SARS-CoV-2 that act by suppressing M^pro^ enzymatic activity [54, 55]. The findings of these studies and ours indicate that echinocandins exert antiviral effects on SARS-CoV-2. Therefore, in addition to antifungal functions, echinocandins may be developed as drugs that act against broad-spectrum viruses. So far, previous studies have described that the antifungals like polyenes and azoles also showed in vitro antiviral activities to inhibit infection of different viruses [50]. For example, the well-known, highly effective polyene, amphotericin B, exhibits antiviral capacity against viruses, such as human immunodeficiency virus, Japanese encephalitis virus, HSV, and vesicular stomatitis virus [50]. Although the mechanism of the antiviral properties of amphotericin B still remains unclear, its inhibitory effect by targeting viral RNA-dependent RNA polymerase was speculated [56]. Itraconazole, one of the triazoles, also showed effective antiviral activity against SARS-CoV-2 [57, 58], influenza virus [59], Ebola virus [60], and parechovirus [61]. In addition to inhibiting the ergosterol biosynthesis, itraconazole exhibits multiple functional properties relating to its antiviral use, such as directly interacting with endolysosomal cholesterol transporter (the Niemann-Pick disease, type C1 protein, NPC1), interfering with oxysterol binding protein 1 (OSBP) and other proteins belonging to the OSBP-related proteins family, and targeting the mammalian target of rapamycin (mTOR) and hedgehog signaling pathways [50]. These revealed that these antifungals pose functions more than suppressing the fungal cell membrane formation and could be repurposed the antiviral interventions. This is similar to enchinocandins like anidulafungin and micafungin, which showed antiviral activity to impair virus entry more than to disrupt fungal cell wall formation.

Thus far, very few studies have investigated whether echinocandins are being used to clinically treat viral infections, which is also the limitation of the current study. We tried to perform a clinical retrospective investigation on hospitalized SFTS patients but failed to find any cases infected with SFTSV combined with fungal infection being administrated with anidulafungin. Recently, micafungin was administered to a patient with COVID-19 accompanied by candidemia and improved the outcome of the patient administered tocilizumab [62]. Anidulafungin and micafungin were the most active antifungal drugs that were used to combat *Candida* isolates recovered from the oropharyngeal lesions of hospitalized COVID-19 patients [63]. These indicate the clinical importance of echinocandins in the treatment of infectious viral diseases, such as COVID-19, compounded by invasive fungal infections. Although detailed molecular mechanisms underlying the antiviral effects of anidulafungin remain to be clarified, the clinical use of antiviral effects of echinocandins against infectious viral diseases deserves to be further investigated.

## Conclusions

Our study revealed the antiviral activity of anidulafungin against SFTSV both in vivo and in vitro and demonstrated the broad-spectrum nature of its antiviral effects against the entry of SARS-CoV-2, SFTSV-related viruses (GTV and HRTV), CCHFV, ZIKV, and HSV-1 into cells. Moreover, in addition to revealing the antifungal activity of echinocandins, our findings suggested the clinical significance of these compounds in treating viral infectious diseases. Therefore, investigating the potential therapeutic effects of echinocandins, such as anidulafungin and micafungin, in treating SFTS and other infectious viral diseases, may promote the design of therapeutic strategies and help improve the treatment and outcomes of patients suffering from serious viral and fungal infections.

## Supplementary Information

**Additional file 1.** Supporting information.

**Additional file 2.** The NC3Rs ARRIVE guidelines 2.0 author checklist for reporting animal research.

**Additional file 3: Table S1.** The IC_50_, IC_90_, CC_50_, and SI values of six FDA-approved drugs that inhibit SFTSV infection. **Table S2.** The white blood cell counts and platelet counts in blood of the SFTSV-challenged (1 LD_50_) A129 mice administrated vehicle or anidulafungin. **Table S3.** The IC_50_, IC_90_, and SI values of anidulafungin that inhibits SARS-CoV-2, GTV, HRTV, ZIKV, CCHFV, and HSV-1 entry into Vero cells.

**Additional file 4: Figure S1.** Quantitative analyses of the expression levels of SFTSV proteins in Vero cells treated with vehicle (DMSO) or anidulafungin. **Figure S2.** IFAs were performed to investigate the inhibitory effects of anidulafungin on SFTSV infection using cell lines of Vero, Huh7, and HEK293, and the effects of two analogs of anidulafungin, micafungin and caspofungin, on SFTSV infection in Vero cells. **Figure S3.** The intracellular distributions of Rab5 and Rab7 in Vero cells treated with or without anidulafungin at indicated time points post SFTSV incubation. **Figure S4.** Measurement of endosomal pH values in cells incubated with SFTSV while treated with or without anidulafungin. **Figure S5.** The percent of survivals and body weight changes monitored for 14 days among the A129 mice challenged by SFTSV doses of 10 LD_50_ and with the treatment of anidulafungin or vehicle.

**Additional file 5.** Original images of Western blots in Fig. 2D, Fig. 3D, Fig. 3E, Fig. 3G, and Fig. 4B, accompanied with brief instructions. 

## Data Availability

Data sharing is not applicable to this article as no datasets were generated or analyzed during the current study.

## Abbreviations

CC_50_: 50% cytotoxic concentration
CCHFV: Crimean-Congo hemorrhagic fever virus
COVID-19: Coronavirus disease 2019
CPZ: Chlorpromazine
DMSO: Dimethyl sulphoxide
GTV: Guertu virus
H&E: Hematoxylin and eosin
HRTV: Heartland virus
HSV-1: Herpes simplex virus 1
IC_50_: 50% inhibitory concentration
IFA: Immunofluorescence assay
IPA3: p21-activated kinase inhibitor III
LD_50_: Median lethal dose
MOI: Multiplicity of infection
NP: Nucleoprotein
PBS: Phosphate buffer saline
SARS-CoV-2: Severe acute respiratory syndrome coronavirus 2
SFTS: Severe fever with thrombocytopenia syndrome disease
SFTSV: Severe fever with thrombocytopenia symdrome virus
SI: Selectivity index
SPF: Specific pathogen-free
ZIKV: Zika virus
