# Supplementary material for: Antiviral activity and mechanism of the antifungal drug, anidulafungin, suggesting its potential to promote treatment of viral diseases
Source: BMC Med. 2022 Oct 21;20:359. doi: 10.1186/s12916-022-02558-z (PMC9585728; doi:10.1186/s12916-022-02558-z)
Supplement: Supplementary file 1 — Additional file 1. Supporting information. [file 12916_2022_2558_MOESM1_ESM.docx]

Supplementary Methods

**In-house preparation of anti-SFTSV Gc polyclonal antibody**

A fragment of partial sequence of SFTSV glycoprotein open reading frame which encodes the SFTSV Gc fragment from position 563 to 1023 aa was amplified by RT-PCR using the cDNA of SFTSV strain HBMC5 as the template. The PCR products were sequenced and cloned into the plasmid pET-28a fused with His Tag to generate the expression plasmid pET-28a-SFTSV GcP. The protein expression in E.coli BL21 cells was validated by SDS-PAGE. Then protein was purified, mixed with complete Freund's adjuvant (1:1), and inoculated into rabbit for three times, 2 weeks apart. The rabbit anti-serum was obtained 2 weeks after the third inoculation, and its specificity to react with SFTSV Gc was validated by Western blot and IFAs.

**Western blot and quantitative real-time PCR assay**

SFTSV-infected cells with or without anidulafungin treatment were harvested at indicated time points. Total RNA was purified using TRIzol reagent (Invitrogen, Carlsbad, USA) according to the manufacturer’s instructions. Quantitative real-time PCR (qRT-PCR) was performed to determine the number of SFTSV RNA copies in infected cells using primers targeting SFTSV NP, as previously described (29). Western blot analyses were performed to detect SFTSV antigen (NP, NSs, Gn, and Gc) expression in cells and the expression of β-actin as a control, as previously described (30). Viral protein expression levels were quantified by ImageJ and normalized to the levels of β-actin in cells.

**Immunofluorescence assays (IFAs)**

IFAs were conducted to analyse SFTSV protein expression in cells as previously described (30). Briefly, Vero cells were fixed by 4% paraformaldehyde in phosphate buffer saline (PBS, pH7.2) and permeabilized by 0.2% Triton X-100. Then, cells were blocked by PBS containing 5% bovine serum albumin (BSA) overnight or at 37 °C for 2h, and incubated with respective primary antibody followed by the secondary antibody. To analyse viral protein expression on cell surface, the fixed cells, which were not treated by 0.2% Triton X-100, were blocked and incubated with antibodies as described above. Cell nuclei were stained using 4', 6-diamidine-2-phenylindole dihydrochloride (DAPI) (Beyotime, Shanghai, China) according to the instruction. Images were taken by an inverted fluorescence microscope.
