# Supplementary material for: Antiviral activity and mechanism of the antifungal drug, anidulafungin, suggesting its potential to promote treatment of viral diseases
Source: BMC Med. 2022 Oct 21;20:359. doi: 10.1186/s12916-022-02558-z (PMC9585728; doi:10.1186/s12916-022-02558-z)
Supplement: Supplementary file 3 — Additional file 3: Table S1. The IC50, IC90, CC50, and SI values of six FDA-approved drugs that inhibit SFTSV infection. Table S2. The white blood cell counts and platelet counts in blood of the SFTSV-challenged (1 LD50) A129 mice administrated vehicle or anidulafungin. Table S3. The IC50, IC90, and SI values of anidulafungin that inhibits SARS-CoV-2, GTV, HRTV, ZIKV, CCHFV, and HSV-1 entry into Vero cells. [file 12916_2022_2558_MOESM3_ESM.docx]

**Table S1** The IC_50_, IC_90_, CC_50_, and SI values of six FDA-approved drugs that inhibit SFTSV infection

| Compounds | IC_50_ (μM) | IC_90_ (μM) | CC_50_ (μM) | SI |
| --- | --- | --- | --- | --- |
| Anidulafungin | 3.02 | 12.44 | 41.69 | 13.80 |
| Atovaqone | 5.06 | 10.13 | >100 | >10 |
| Mycophenolic acid | 0.47 | 4.75 | >100 | >10 |
| Mycophenolate mofetil | 0.57 | 1.52 | >100 | >10 |
| Nitazoxanide | 11.11 | 25.33 | >100 | >10 |
| Vidofludimus | 6.07 | 14.55 | >100 | >10 |

**Table S2.** The white blood cell counts and platelet counts in blood of the SFTSV-challenged (1 LD_50_) A129 mice administrated vehicle or anidulafungin

| Parameters | SFTSV+Vehicle (median, IQR) | |  | SFTSV+Anidulafungin (median, IQR) | | | Control* |
| --- | --- | --- | --- | --- | --- | --- | --- |
|  | D3 | D7 |  | D3 | D7 | D14 |  |
| WBC (×10^9^/L) | 6.52  (6.5, 8.0) | 6.25  (5.535, 7.085) |  | 4.75  (4.35, 5.465) | 4.72  (4.31, 6.195) | 5.76  (5.28, 6.04) | 6.79  (5.765, 8.07) |
| PLT (×10^9^/L) | 1075  (1032, 1083) | 992.5  (849.5, 1050.75) |  | 1054.5  (993.25, 1094) | 950  (494.5, 1062.75) | 1141  (1050, 1191) | 2493.5  (2403, 2563) |

* Control shows the results using the serum samples collected from A129 mice before SFTSV infection (day 0). IQR, the interquartile range.

**Table S3.** The IC_50_, IC_90_, and SI values of anidulafungin that inhibits SARS-CoV-2, GTV, HRTV, ZIKV, CCHFV, and HSV-1 entry into Vero cells

| Virus | IC_50_ (μM) | IC_90_ (μM) | SI |
| --- | --- | --- | --- |
| SARS-CoV-2 | 5.49 | 8.55 | 7.59 |
| GTV | 5.54 | 9.62 | 7.53 |
| HRTV | 4.99 | 9.29 | 8.35 |
| ZIKV | 4.37 | 6.44 | 9.54 |
| CCHFV | 10.80 | 24.22 | 3.86 |
| HSV-1 | 6.81 | 9.12 | 6.12 |
