# Supplementary material for: Antiviral activity and mechanism of the antifungal drug, anidulafungin, suggesting its potential to promote treatment of viral diseases
Source: BMC Med. 2022 Oct 21;20:359. doi: 10.1186/s12916-022-02558-z (PMC9585728; doi:10.1186/s12916-022-02558-z)
Supplement: Supplementary file 4 — Additional file 4: Figure S1. Quantitative analyses of the expression levels of SFTSV proteins in Vero cells treated with vehicle (DMSO) or anidulafungin. Figure S2. IFAs were performed to investigate the inhibitory effects of anidulafungin on SFTSV infection using cell lines of Vero, Huh7, and HEK293, and the effects of two analogs of anidulafungin, micafungin and caspofungin, on SFTSV infection in Vero cells. Figure S3. The intracellular distributions of Rab5 and Rab7 in Vero cells treated with or without anidulafungin at indicated time points post SFTSV incubation. Figure S4. Measurement of endosomal pH values in cells incubated with SFTSV while treated with or without anidulafungin. Figure S5. The percent of survivals and body weight changes monitored for 14 days among the A129 mice challenged by SFTSV doses of 10 LD50 and with the treatment of anidulafungin or vehicle. [file 12916_2022_2558_MOESM4_ESM.pdf]

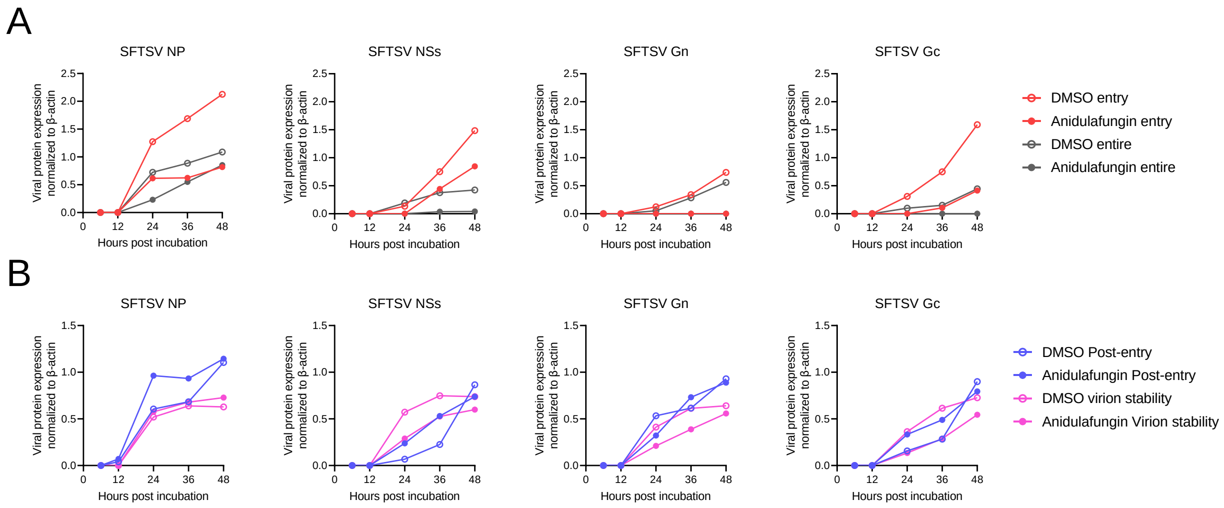


**Figure S1.** Quantitative analyses of the expression levels of SFTSV proteins in Vero cells treated with vehicle (DMSO) or anidulafungin throughout the entire process (A) or in different stages of virus infection including virus entry (A), virion stability (B), and post entry (B) (as shown in Figure 2A) at indicated time points post virus incubation. The protein signals detected by Western blot were quantified by ImageJ. Levels of each viral protein expression were normalized to that of β-actin in cells.

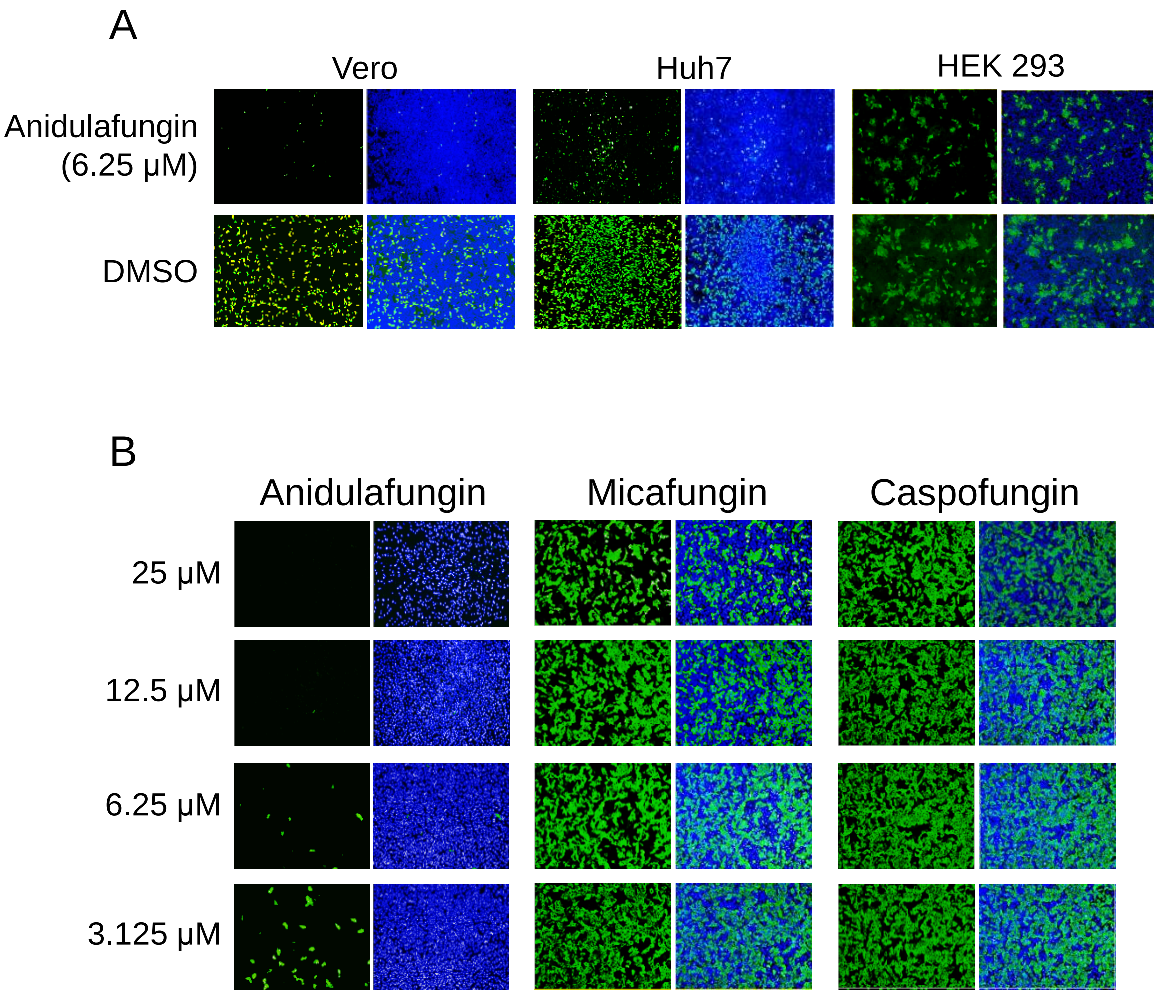


**Figure. S2.** IFAs were performed to investigate (A) the inhibitory effects of anidulafungin on SFTSV infection using cell lines of Vero, Huh7, and HEK293, and (B) the effects of two analogs of anidulafungin, micafungin and caspofungin, on SFTSV infection in Vero cells.

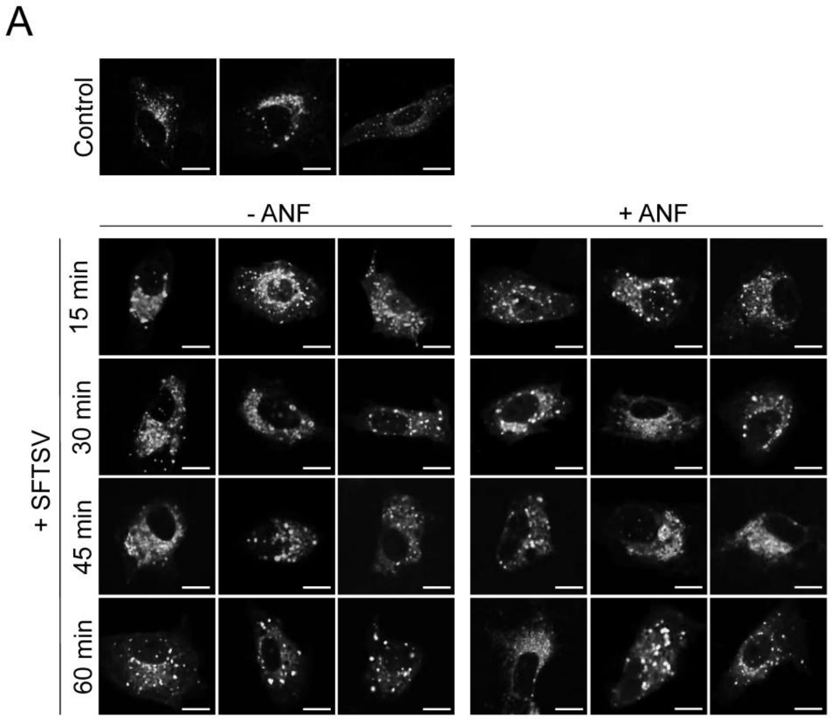
A
Control
- ANF
+ ANF
+ SFTSV
15 min
30 min
45 min
60 min

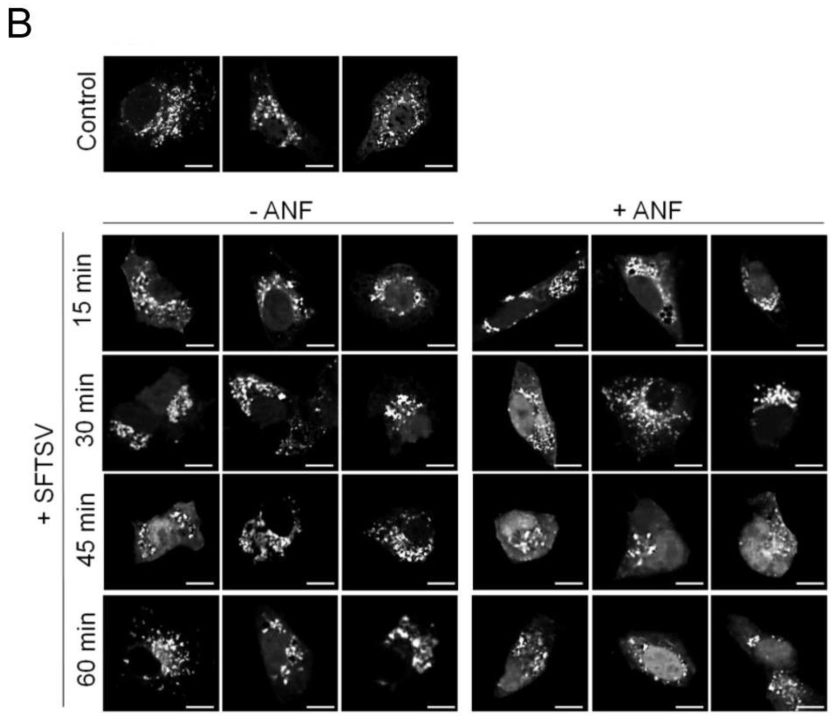
B
Control
- ANF
+ ANF
+ SFTSV
15 min
30 min
45 min
60 min

**Figure. S3.** The intracellular distributions of Rab5 and Rab7 in Vero cells treated with or without anidulafungin at indicated time points post SFTSV incubation. Three images for each test at 10, 30, 45, and 60 min post SFTSV inoculation whilst anidulafungin treatment were shown to present the intracellular location of Rab 5 and Rab7. ANF, anidulafungin. Bars, 5 μm.

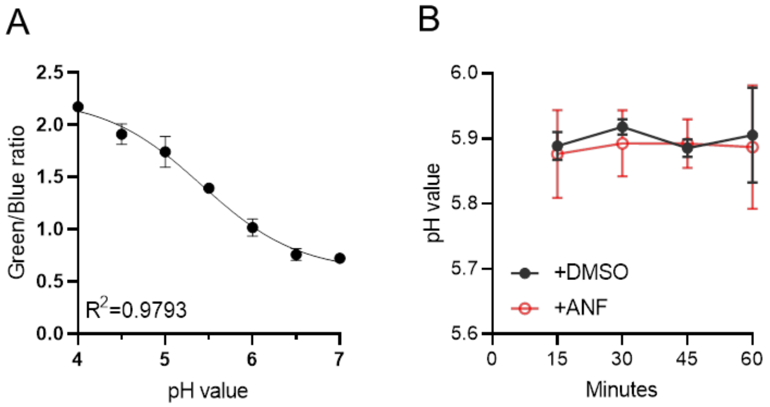


**Figure. S4.** Measurement of endosomal pH values in cells incubated with SFTSV while treated with or without anidulafungin. (A) The standard curve between pH and green/blue ratio fitted by the Boltzmann Sigmoid model. (B) The pH values were calculated based on the standard curve at 15, 30, 45, and 60 min post virus incubation while cells were also treated with anidulafungin or DMSO. ANF, anidulafungin.

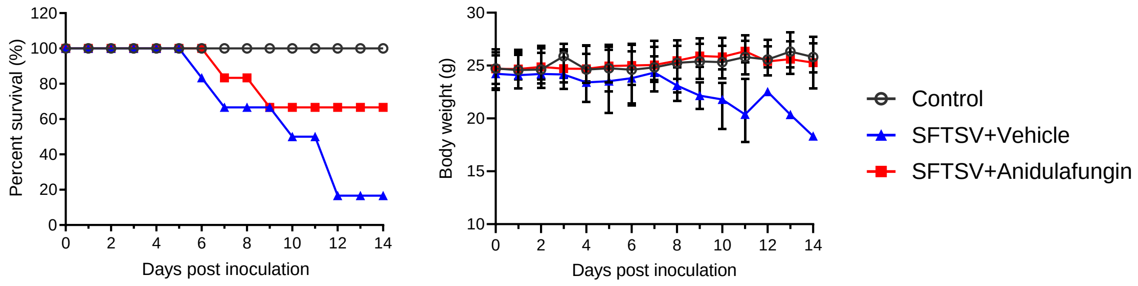

**Figure. S5.** The percent of survivals and body weight changes monitored for 14 days among the A129 mice challenged by SFTSV doses of 10 $LD_{50}$ and with the treatment of anidulafungin or vehicle.
