# Supplementary material for: Antiviral activity and mechanism of the antifungal drug, anidulafungin, suggesting its potential to promote treatment of viral diseases
Source: BMC Med. 2022 Oct 21;20:359. doi: 10.1186/s12916-022-02558-z (PMC9585728; doi:10.1186/s12916-022-02558-z)

**Fig. 2D**

All blots were performed using the cell samples collected at 6, 12, 24, 36, 48, 60, and 72 h post infection. So there are more lanes and bands showing in these images. The bands detected at 60 and 72 h post infection were not shown in Figures.

***Entire stage***

NP (+DMSO)


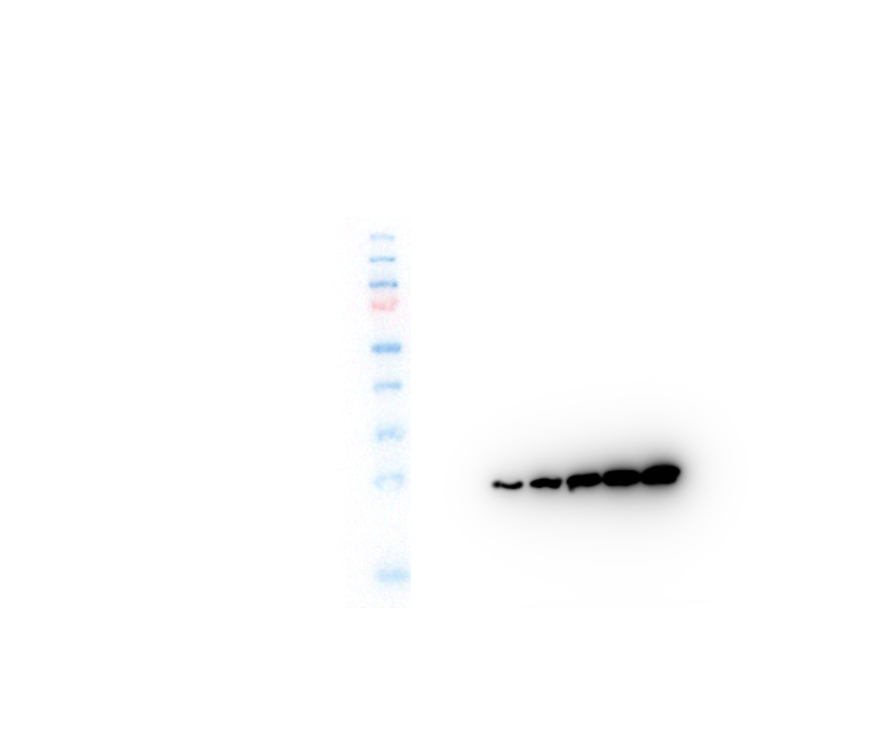


NP (+Anidulafungin, on the right of Marker)


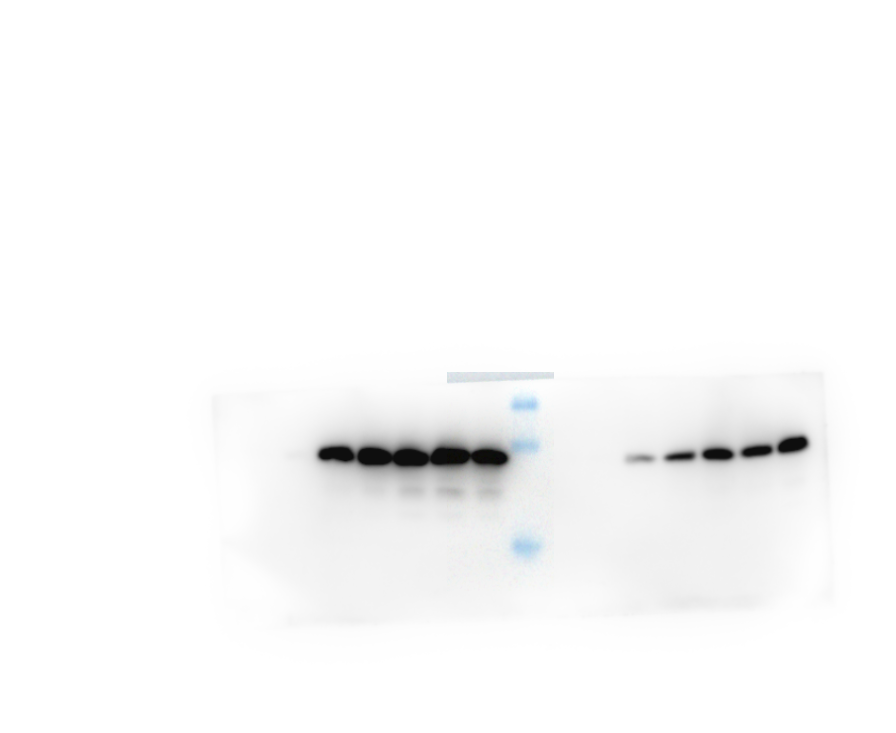


NSs (+ DMSO, left; + Anidulafungin, right)


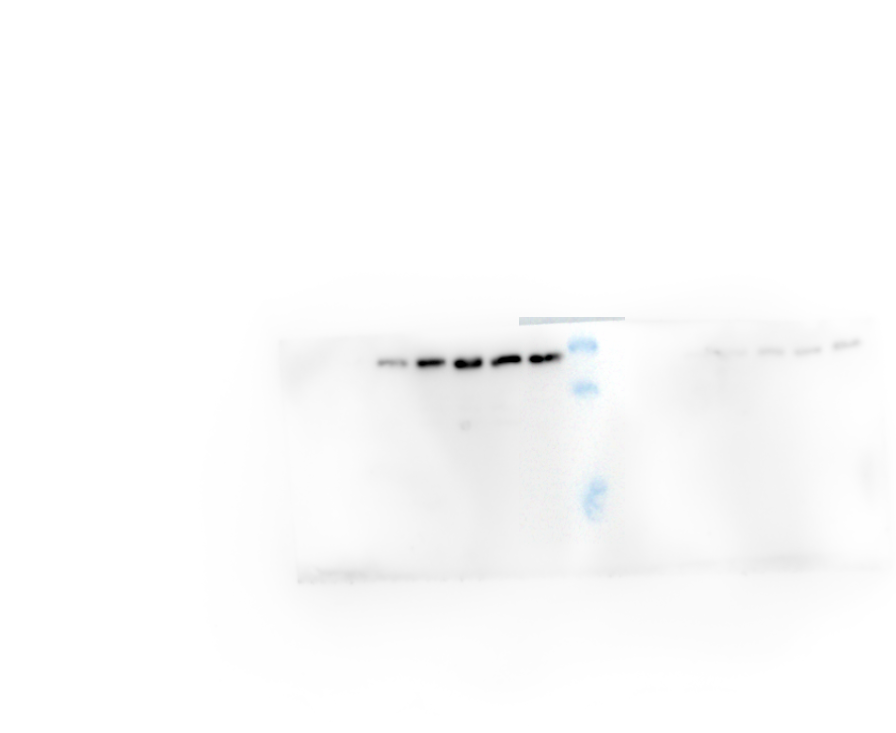


Gn (+ DMSO, left; + Anidulafungin, right)


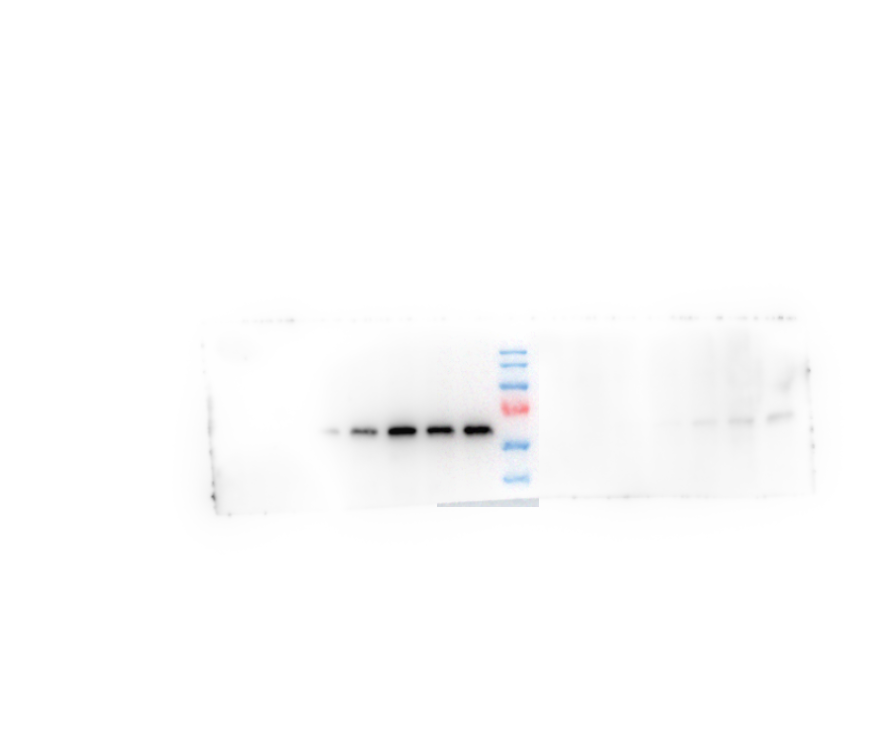


Gc (+ DMSO, left; + Anidulafungin, right)


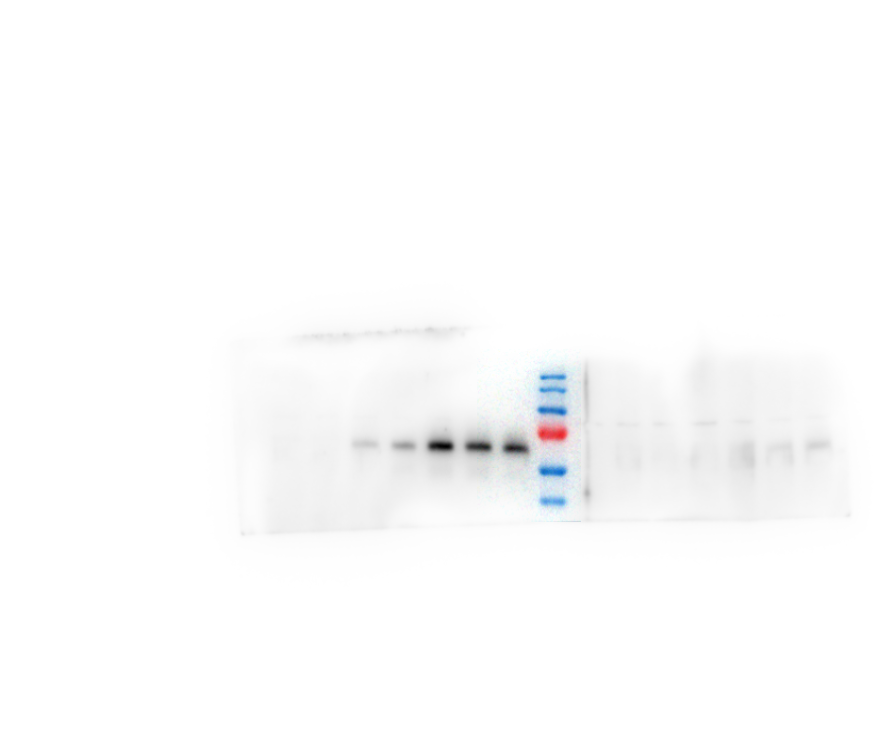


Actin (+ Anidulafungin, right)


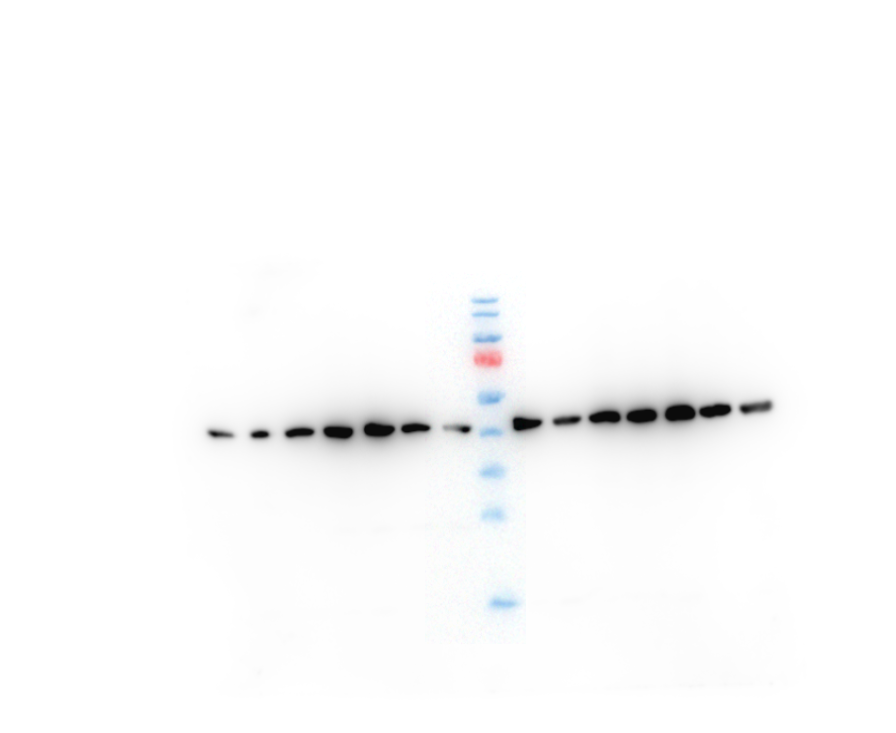


Actin (+ DMSO)


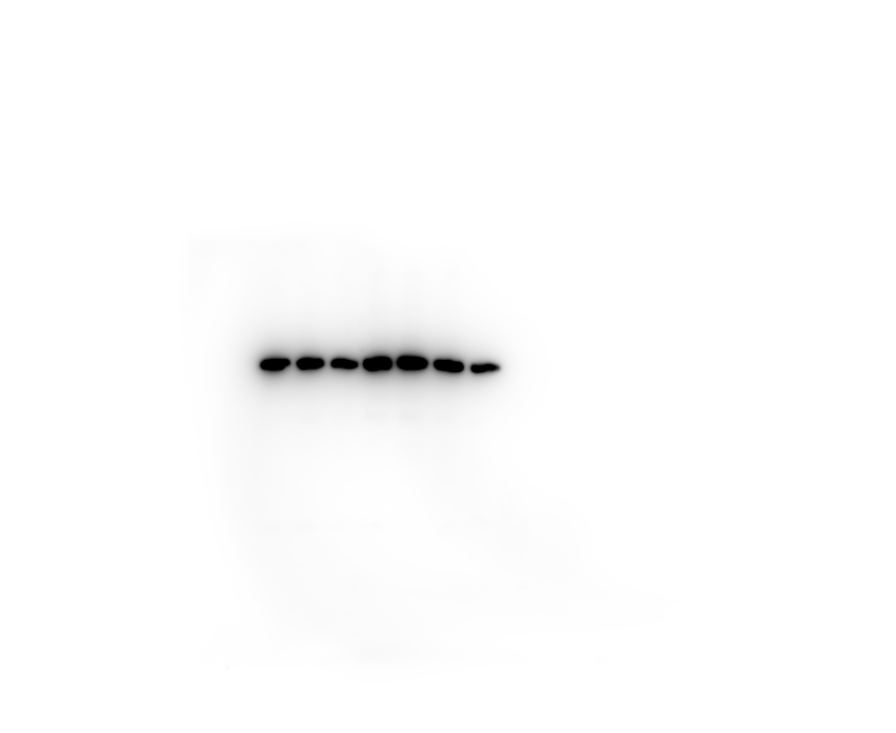


***Virus entry***

NP + Anidulafungin


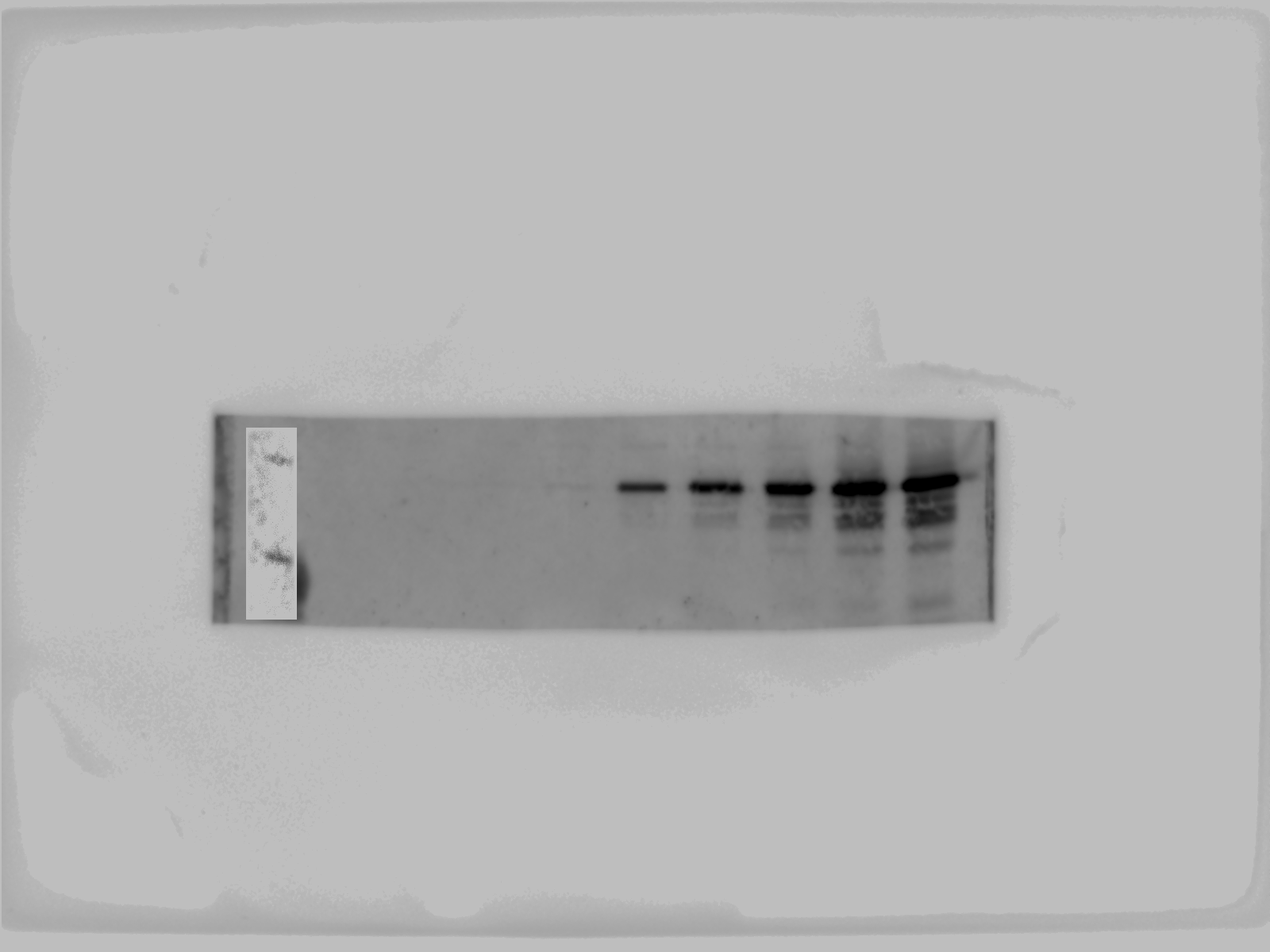


NSs + Anidulafungin


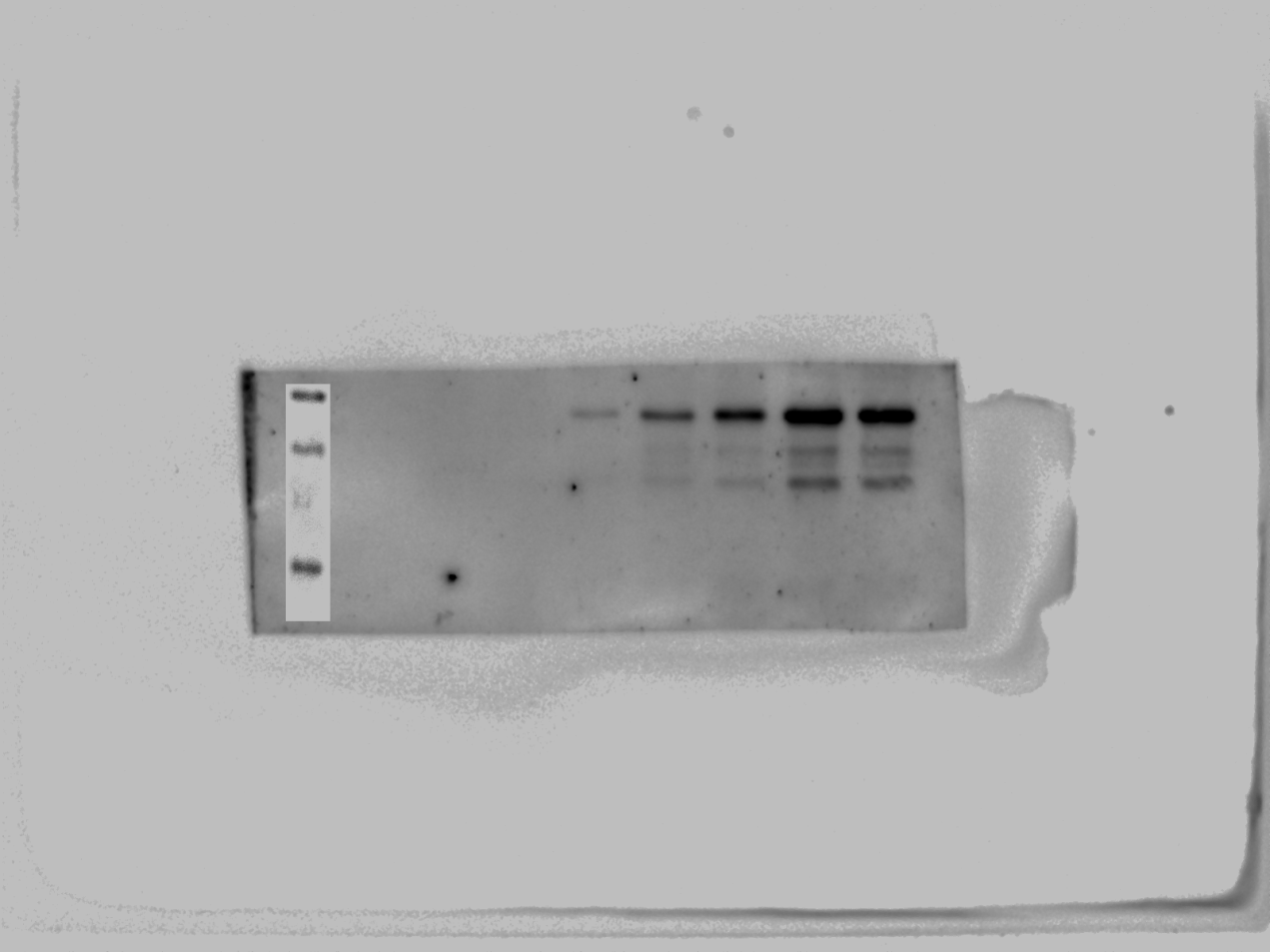


Gn + Anidulafungin


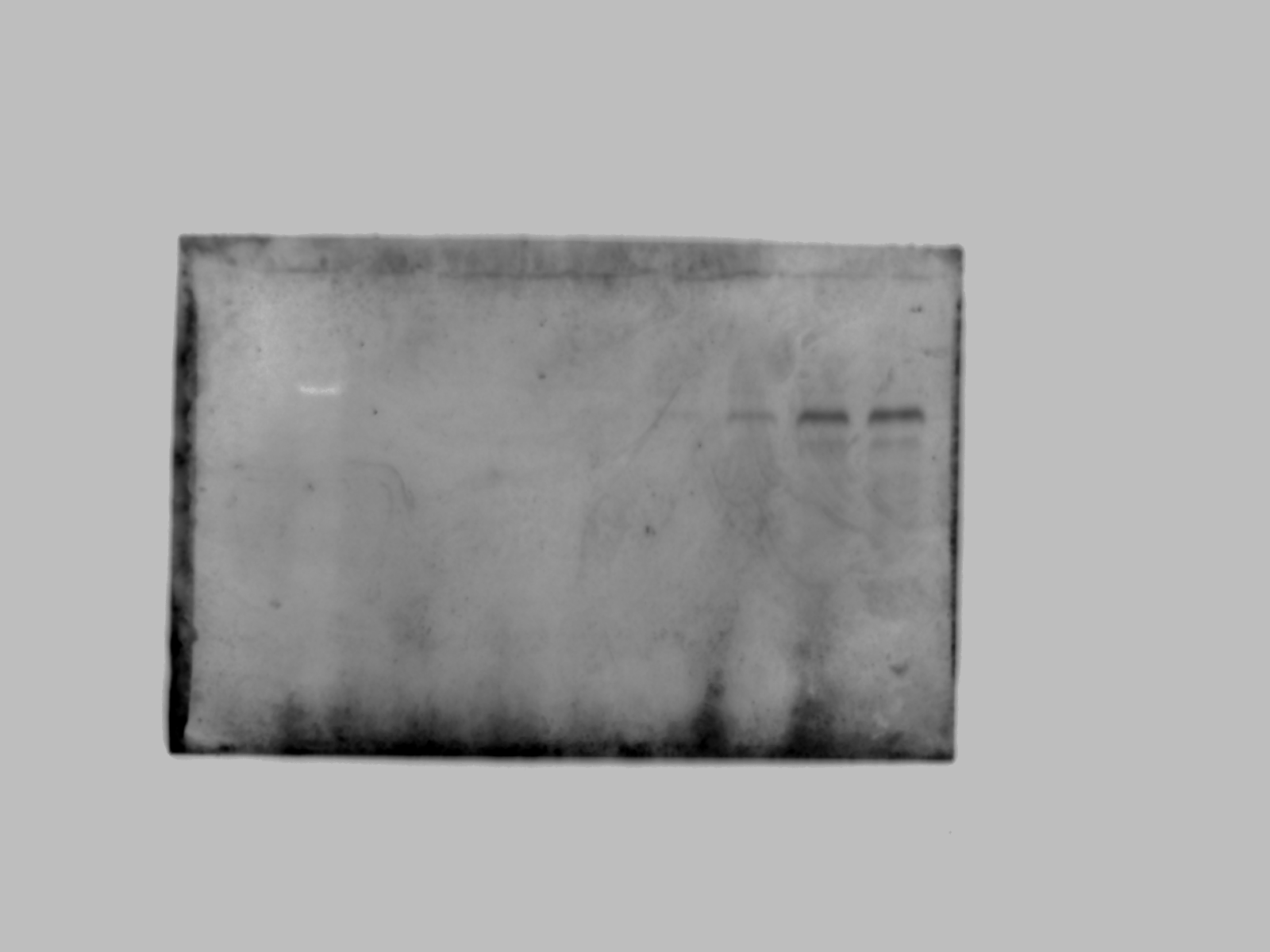


Gc + Anidulafungin


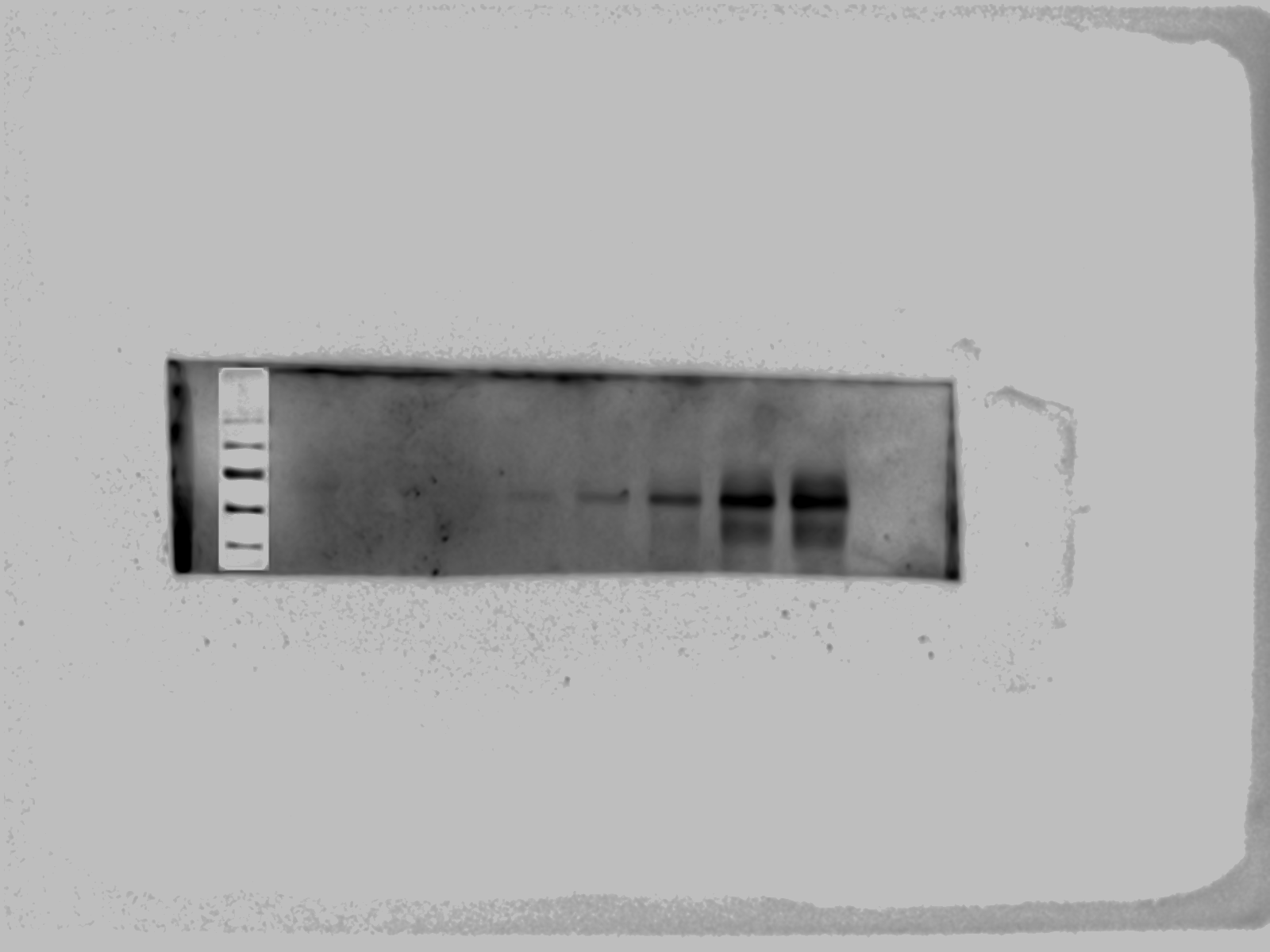


Actin + Anidulafungin


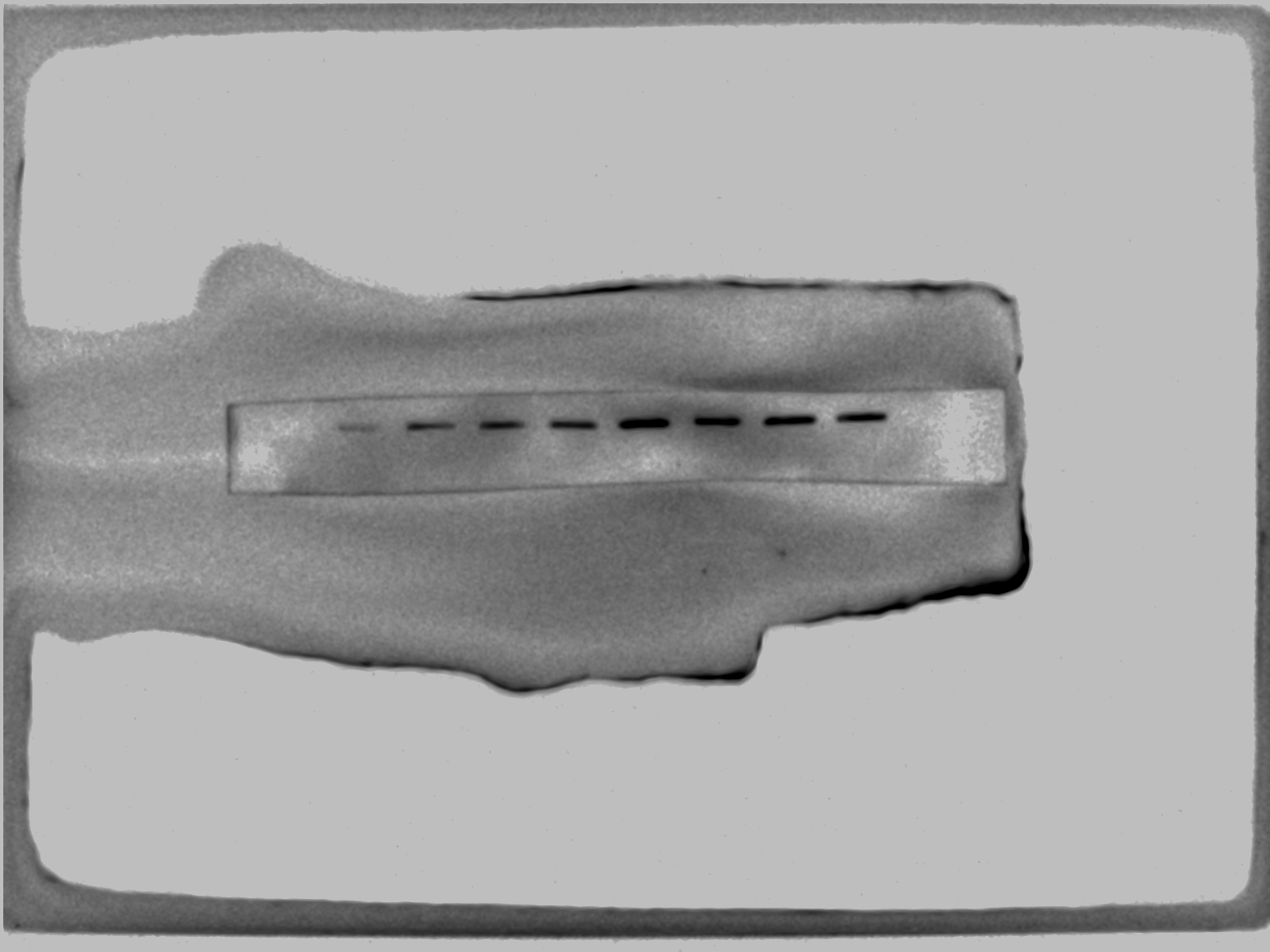


NP +DMSO


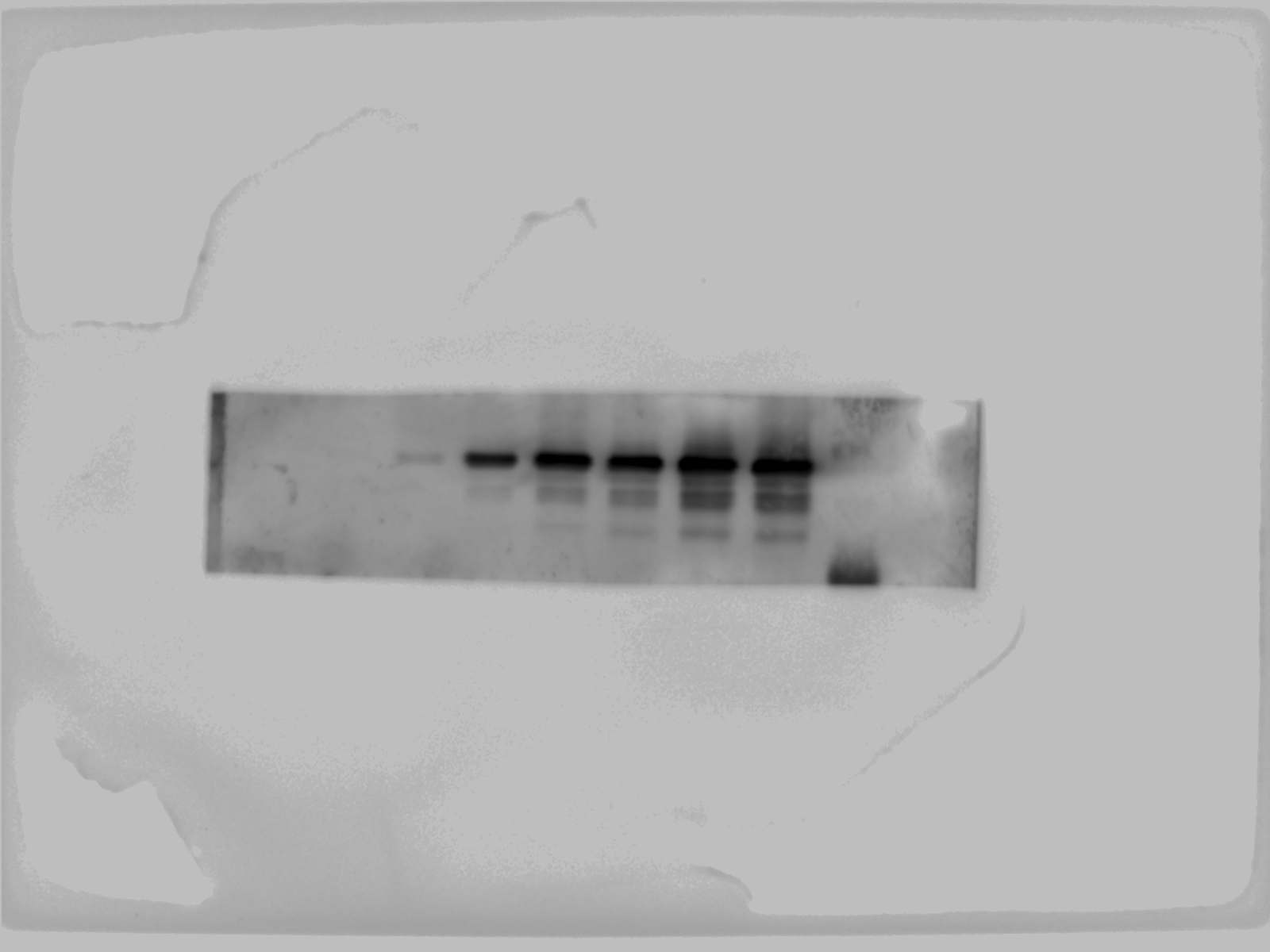


NSs +DMSO


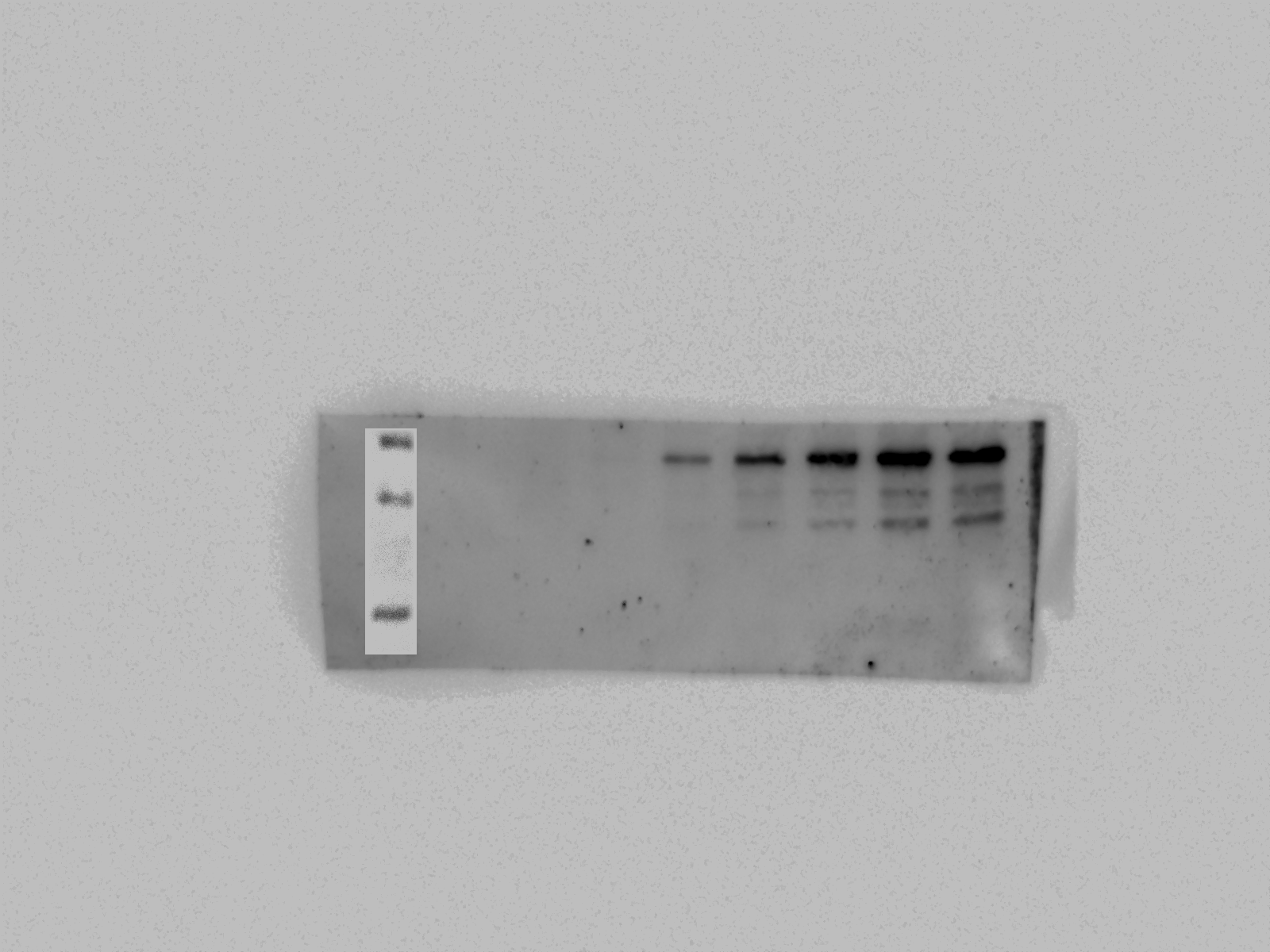


Gn +DMSO


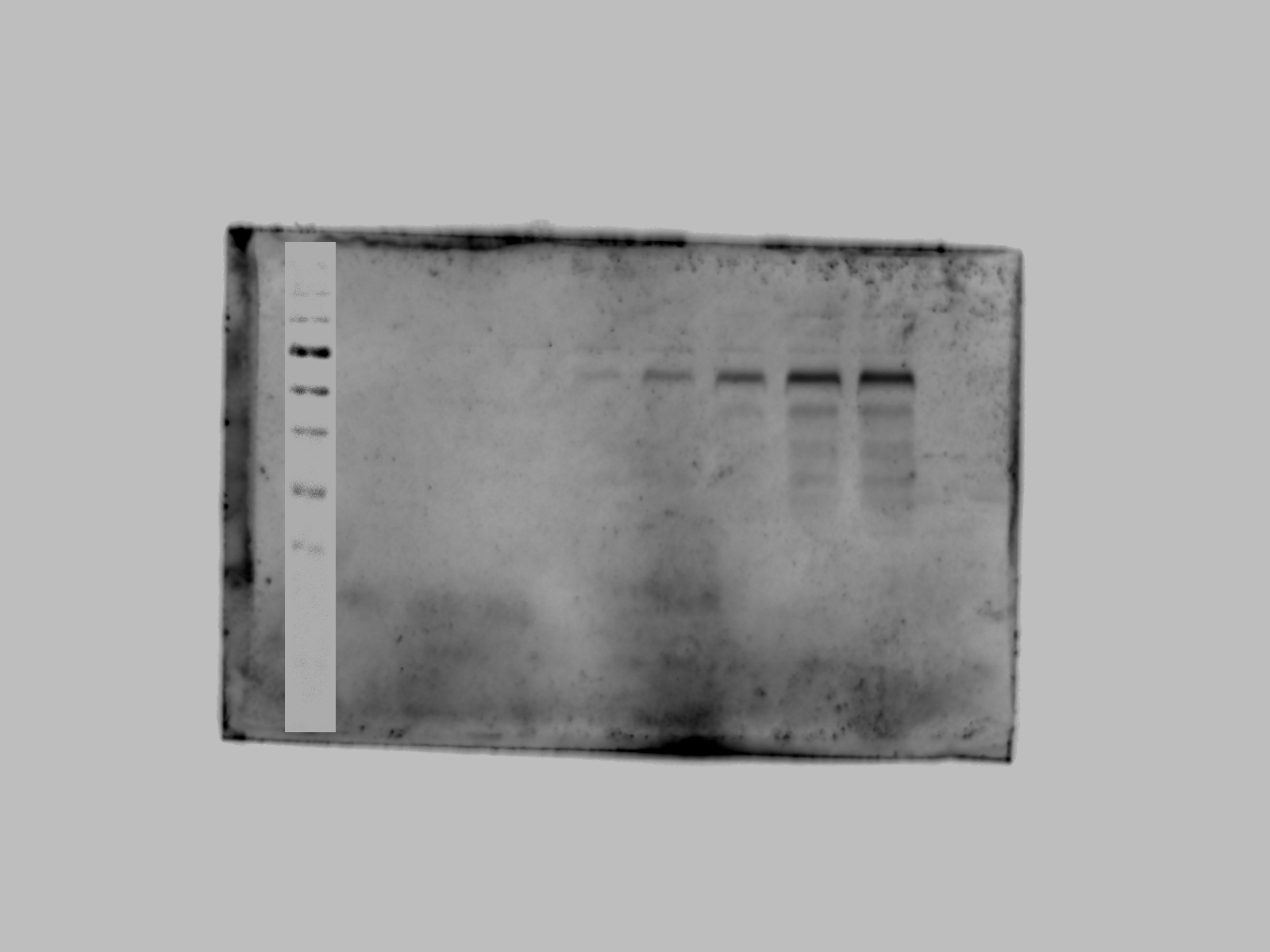


Gc +DMSO


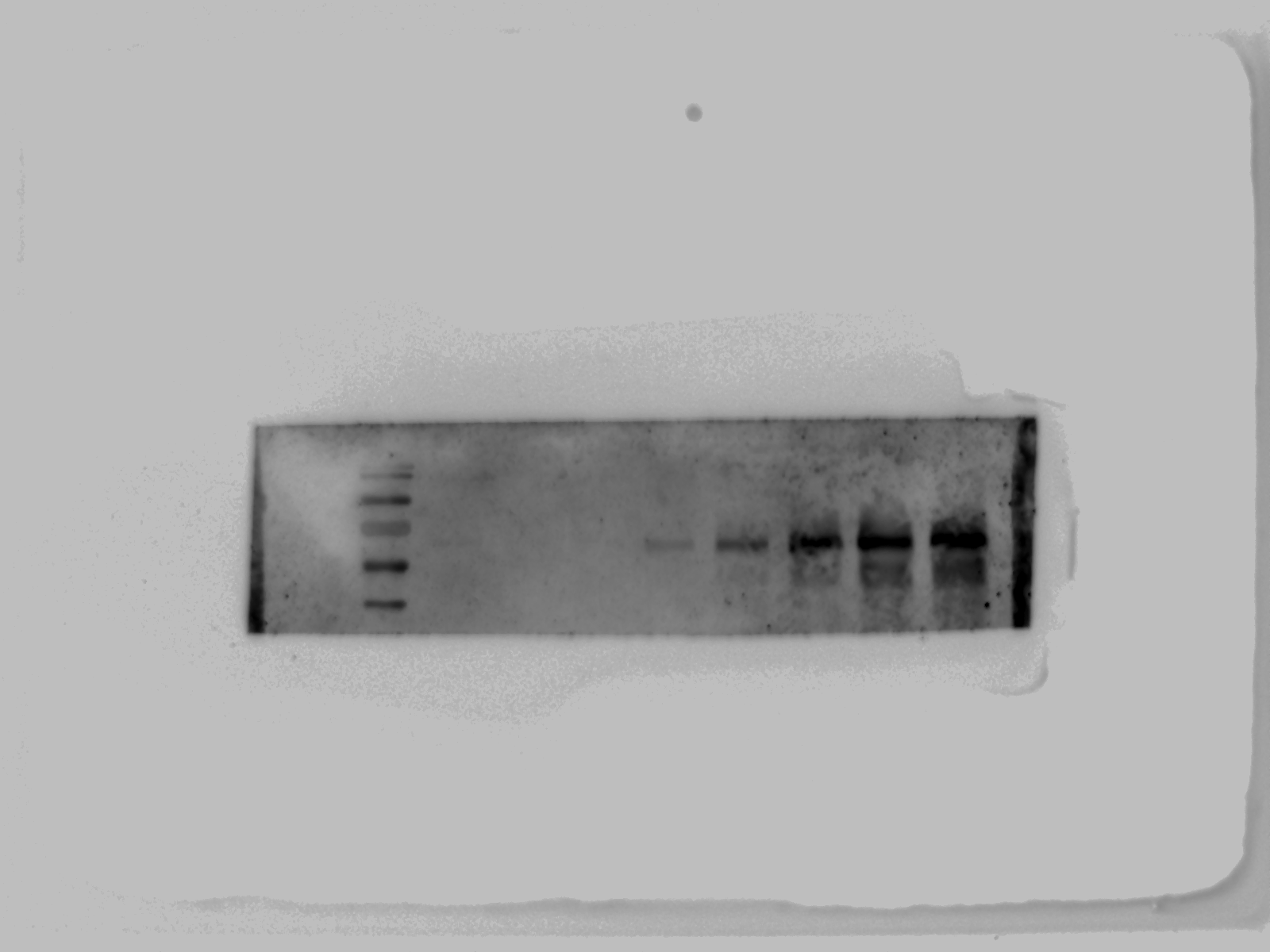


Actin +DMSO


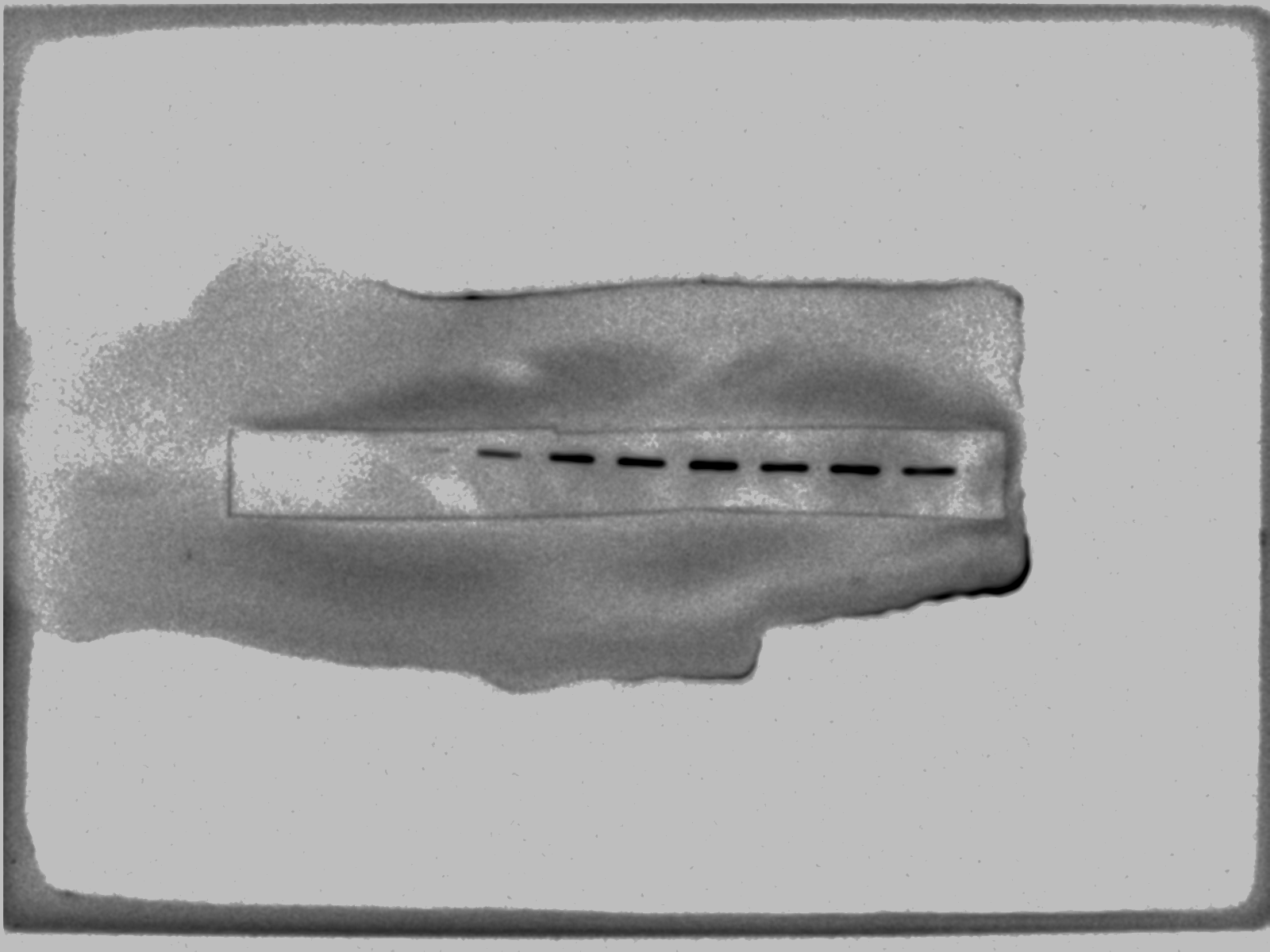


***Virion stability***

NP (+ DMSO, left; + Anidulafungin, right)


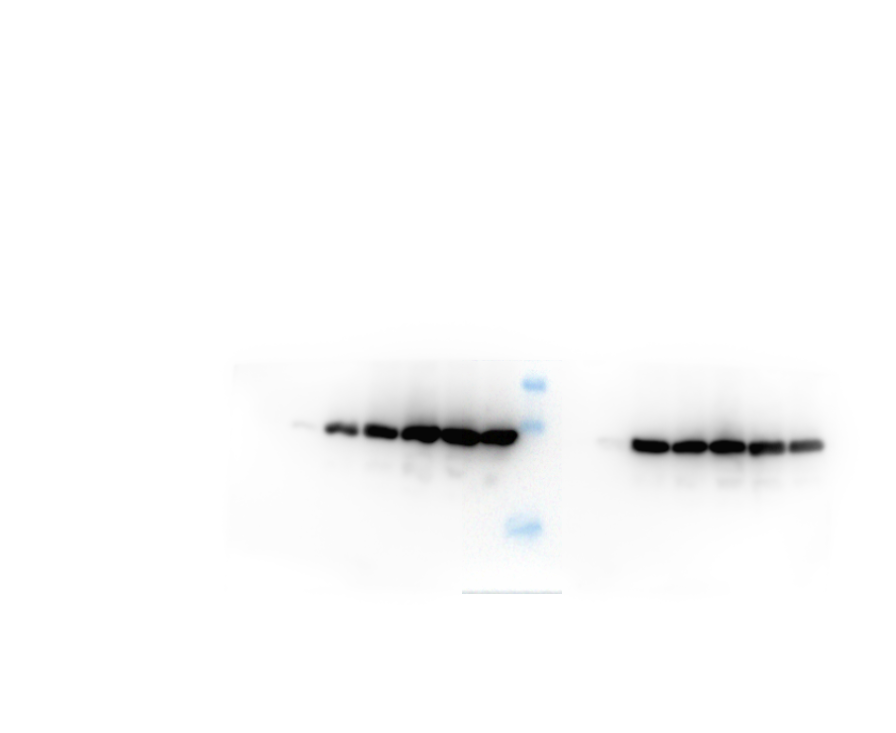


NSs (+ DMSO, left; + Anidulafungin, right)


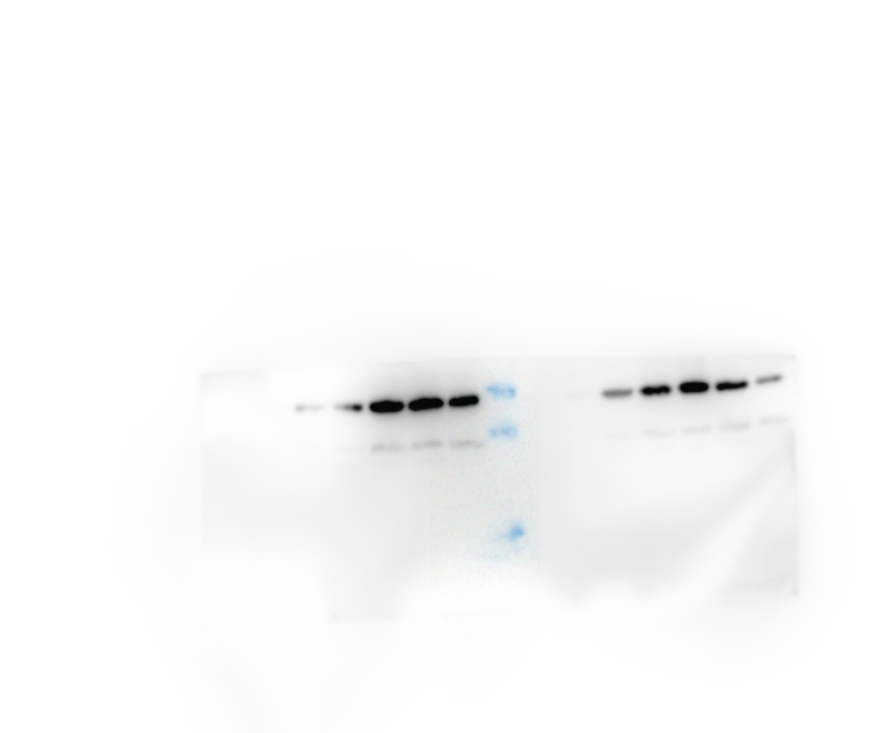


Gn (+ DMSO, left; + Anidulafungin, right)


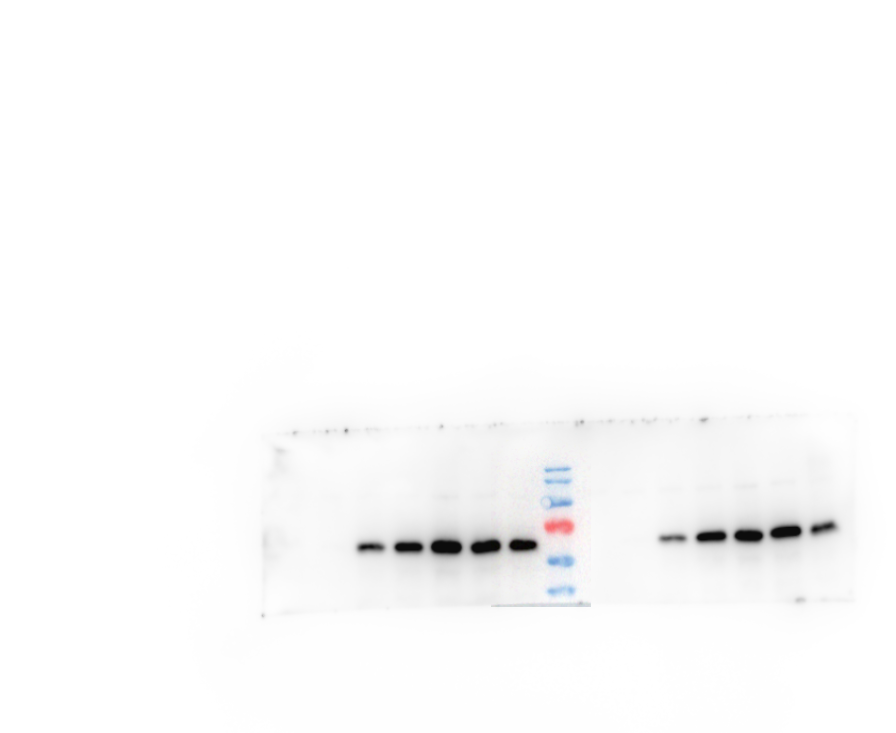


Gc (+ DMSO, left; + Anidulafungin, right)


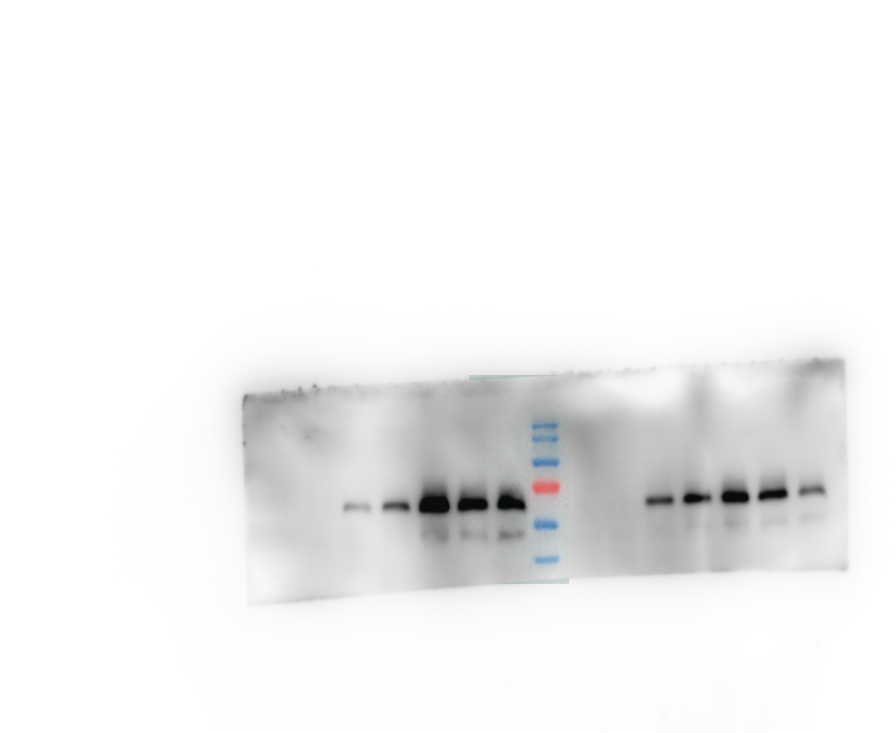


Actin (+ DMSO, left; + Anidulafungin, right)


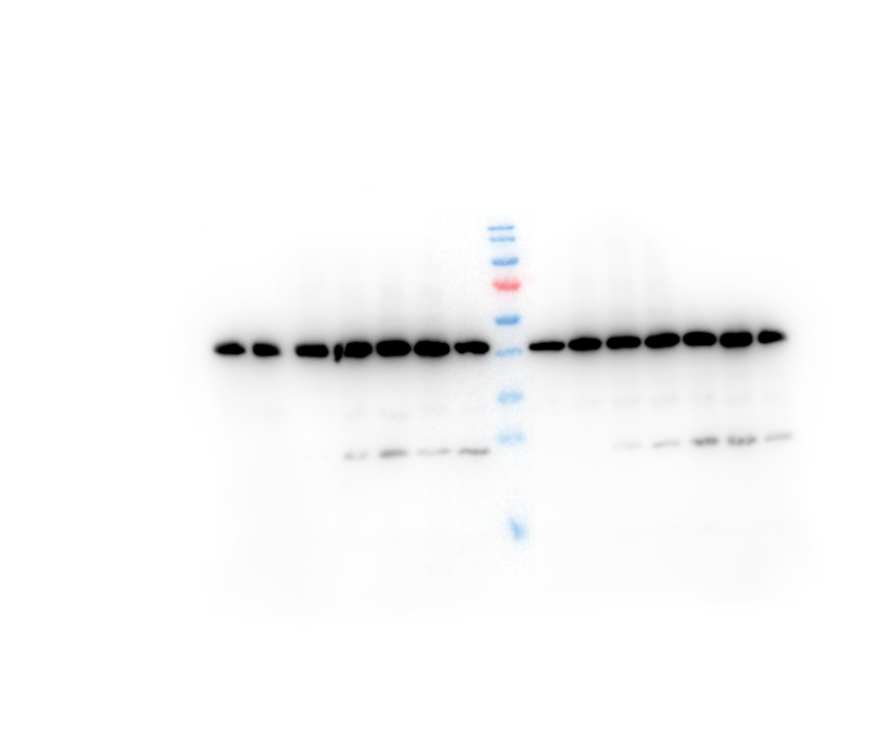


***Post-entry***

NP (+ DMSO, left; + Anidulafungin, right)


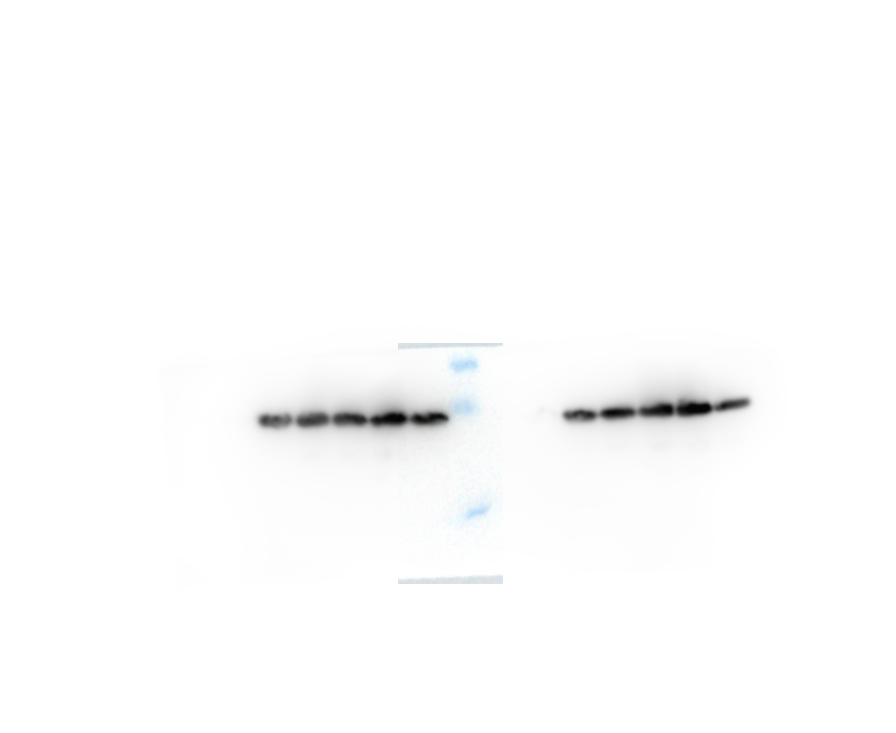


NSs (+ DMSO, left; + Anidulafungin, right)


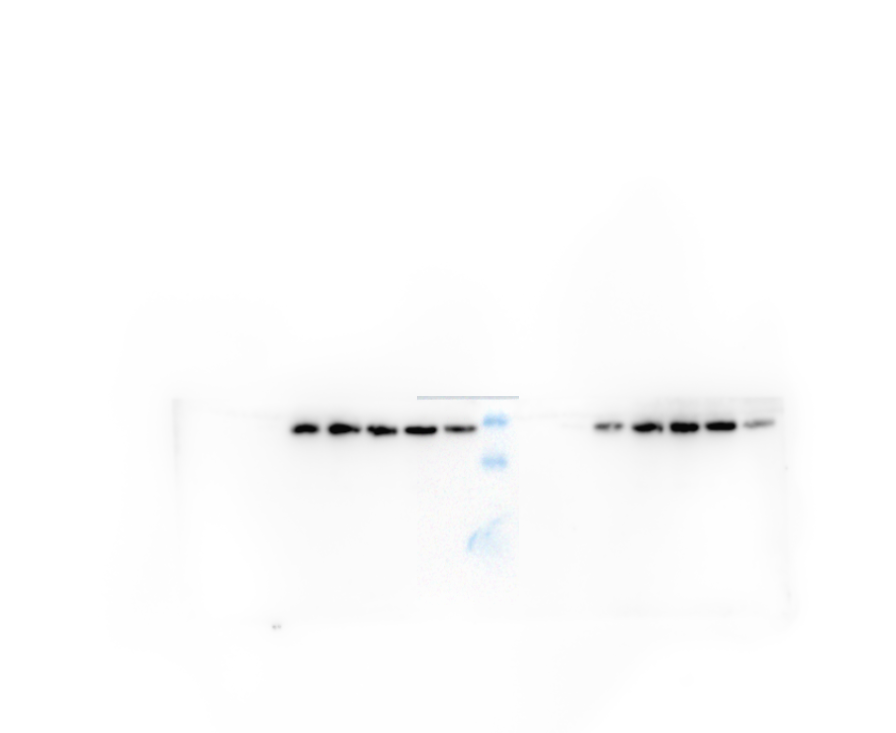


Gn (+ DMSO, left; + Anidulafungin, right)


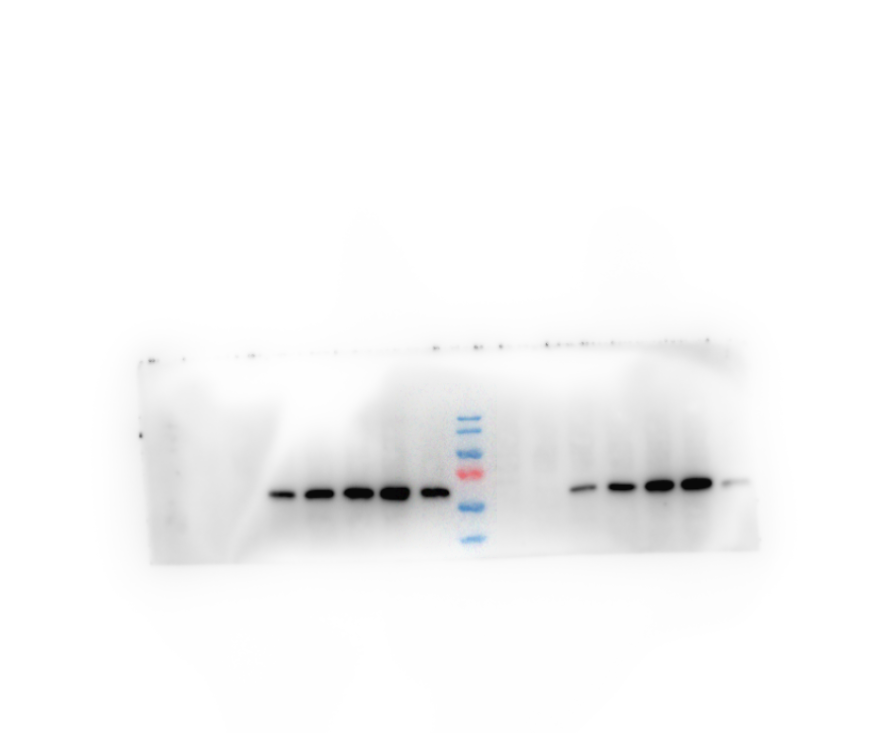


Gc (+ DMSO, left; + Anidulafungin, right)


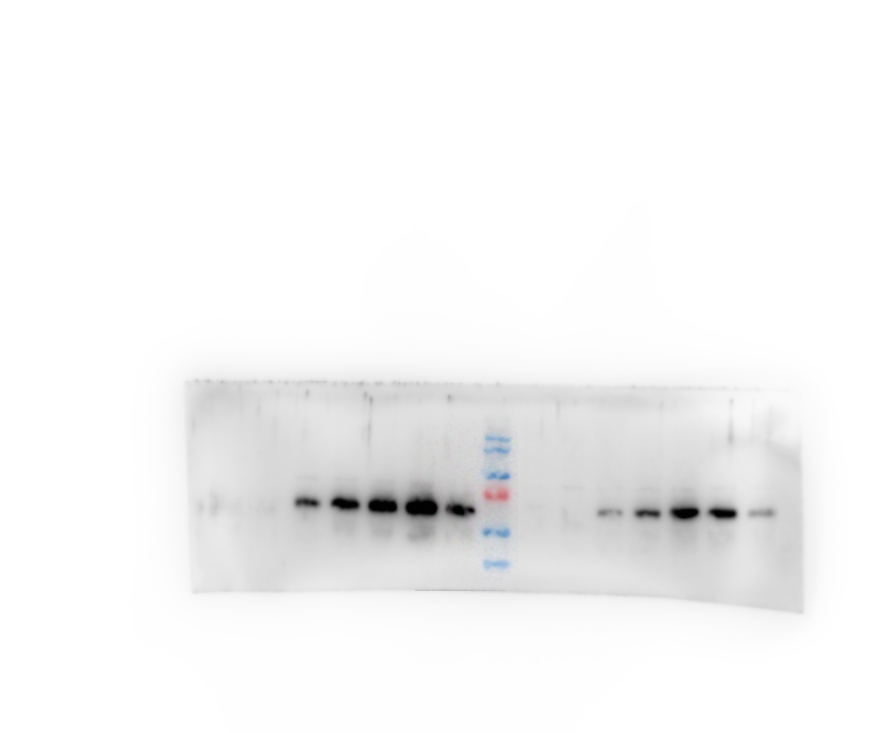


Actin (+ DMSO, left; + Anidulafungin, right)


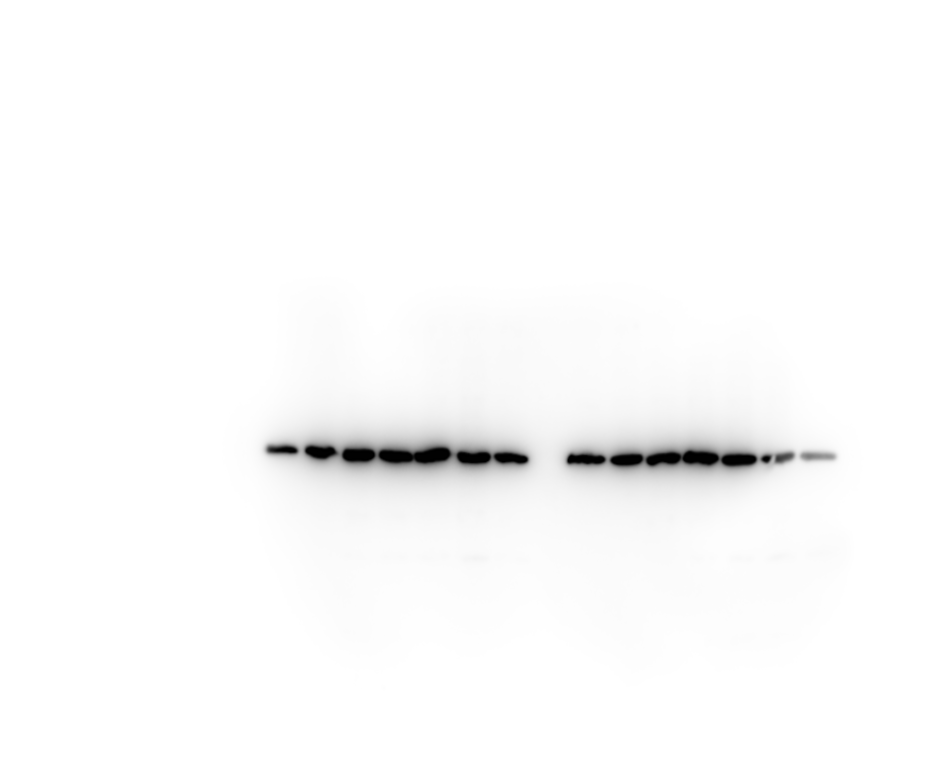


**Fig. 3D**

SFTSV NP (lanes 1-3, (CPZ, Nystatin, and IPA3) - ANF; lanes 4-6, (CPZ, Nystatin, and IPA3) + ANF; lanes 7-9, +ANF, Control, and NC, which are not included in the Figure)


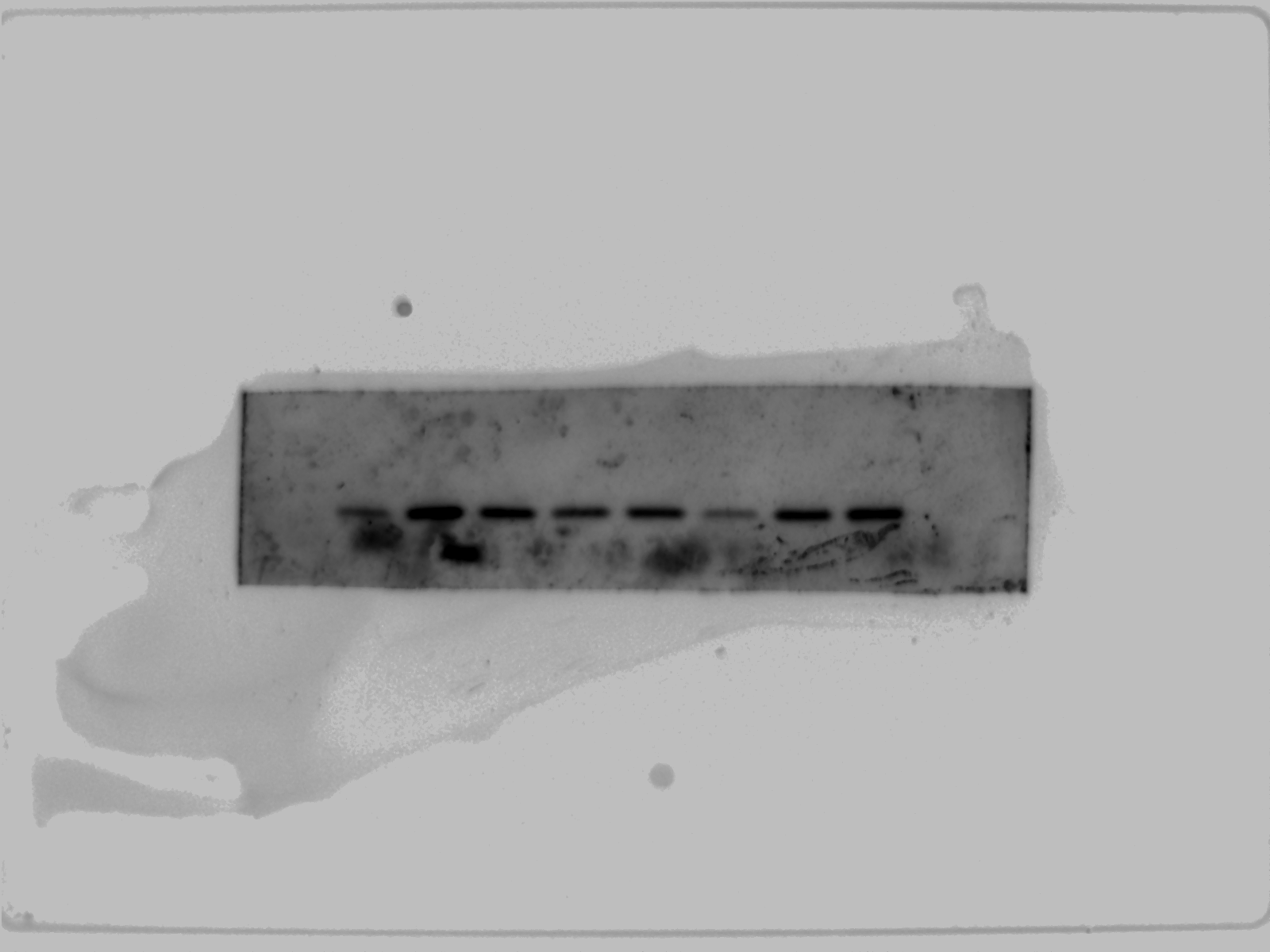


Actin (lanes 1-3, (CPZ, Nystatin, and IPA3) - ANF; lanes 4-6, (CPZ, Nystatin, and IPA3) + ANF; lanes 7-9, +ANF, Control, and NC, which are not included in the Figure)


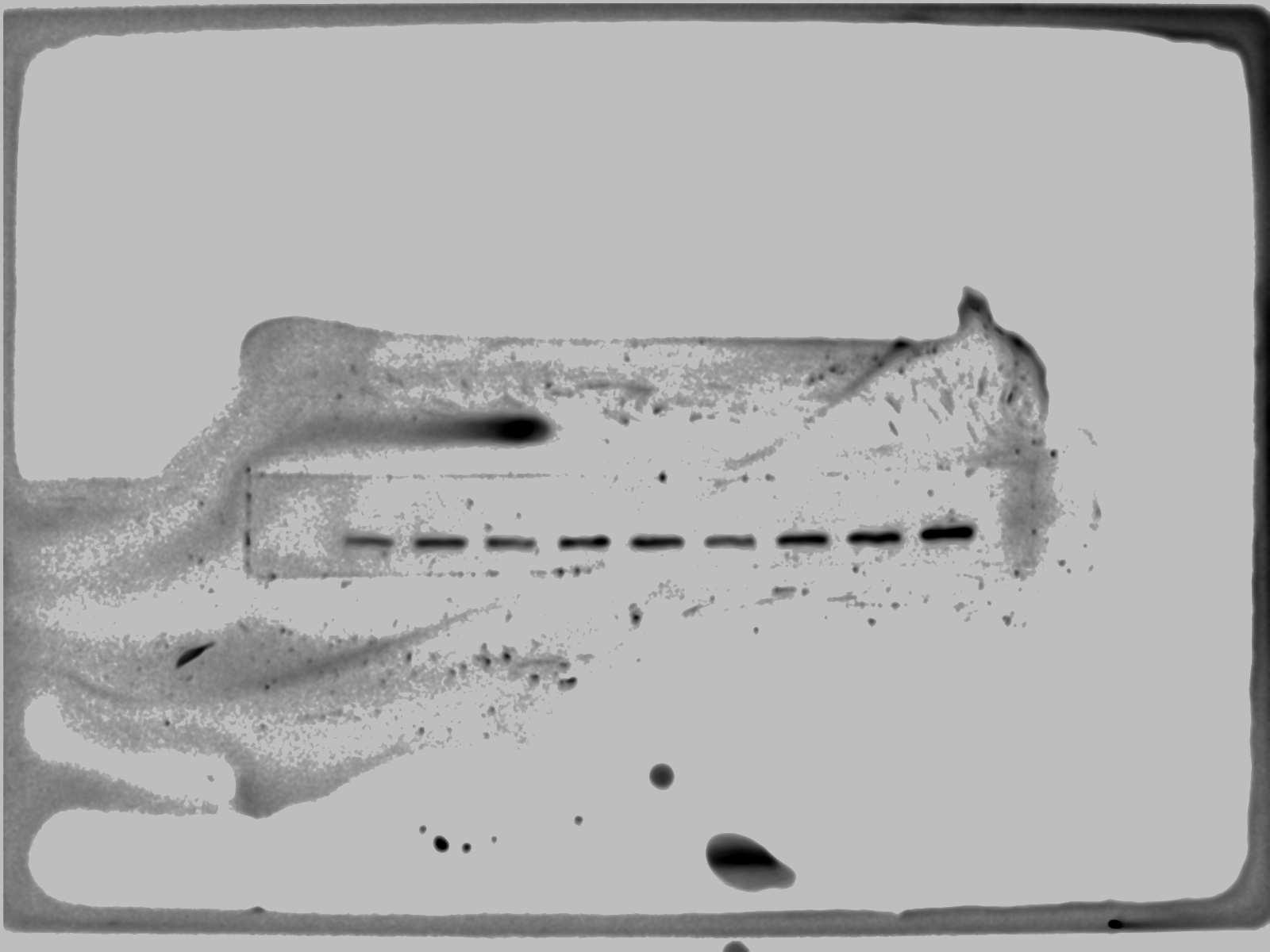


**Fig. 3E**

SFTSV NP


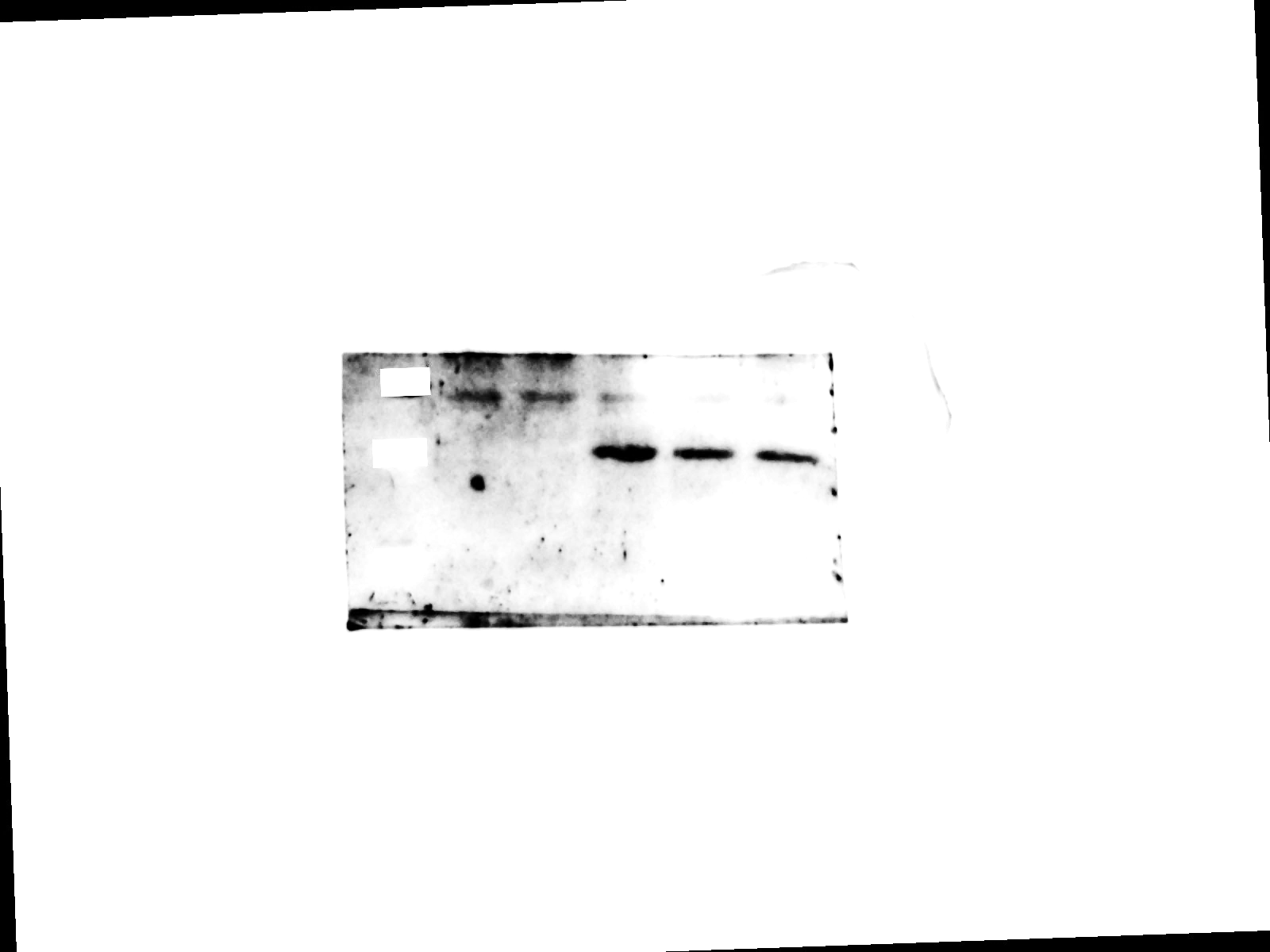


Actin

Actin


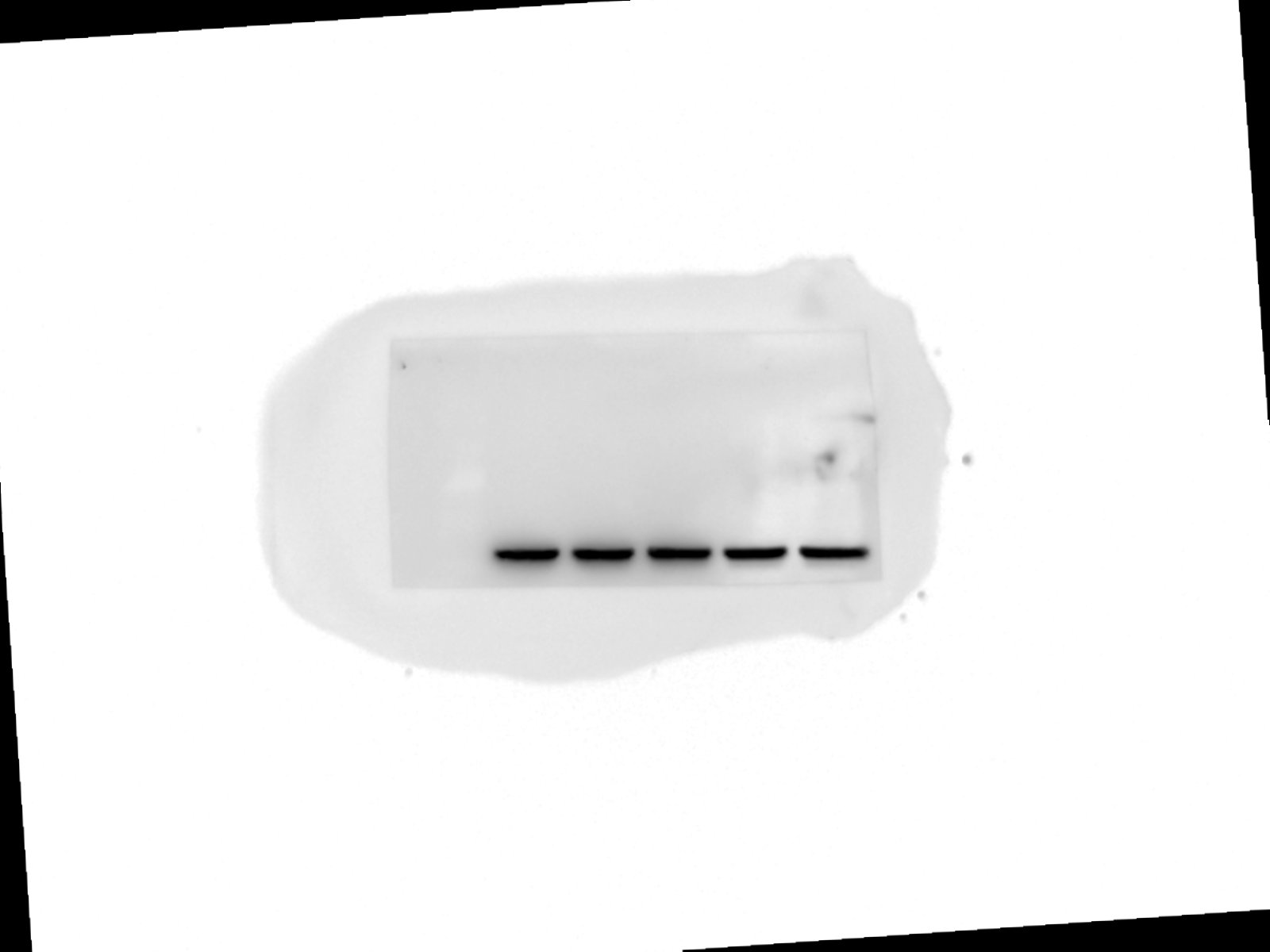


**Fig. 3G**

***Rab 5 transfection*** (lane 1, Marker; other lanes are not included in the study.)

SFTSV NP (lanes 2-5)


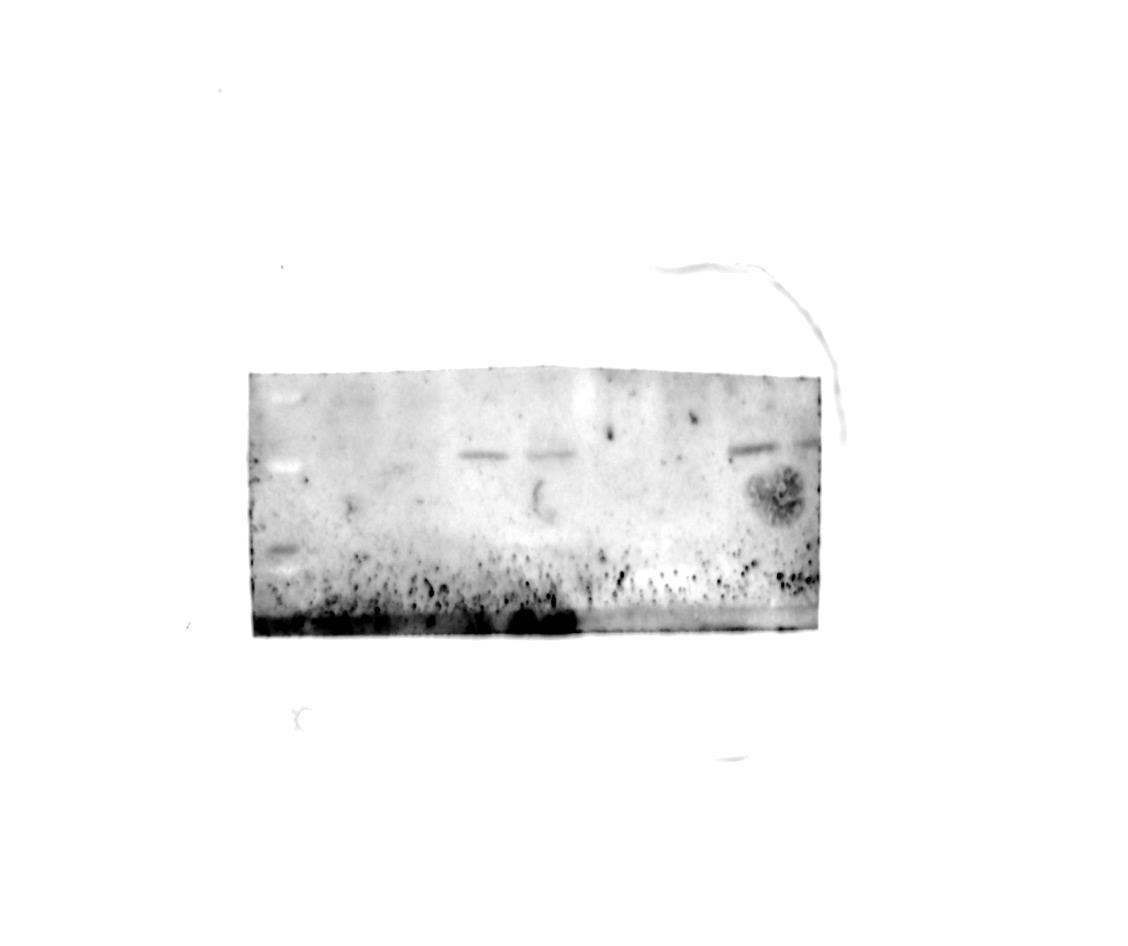


Actin (lanes 2-5)


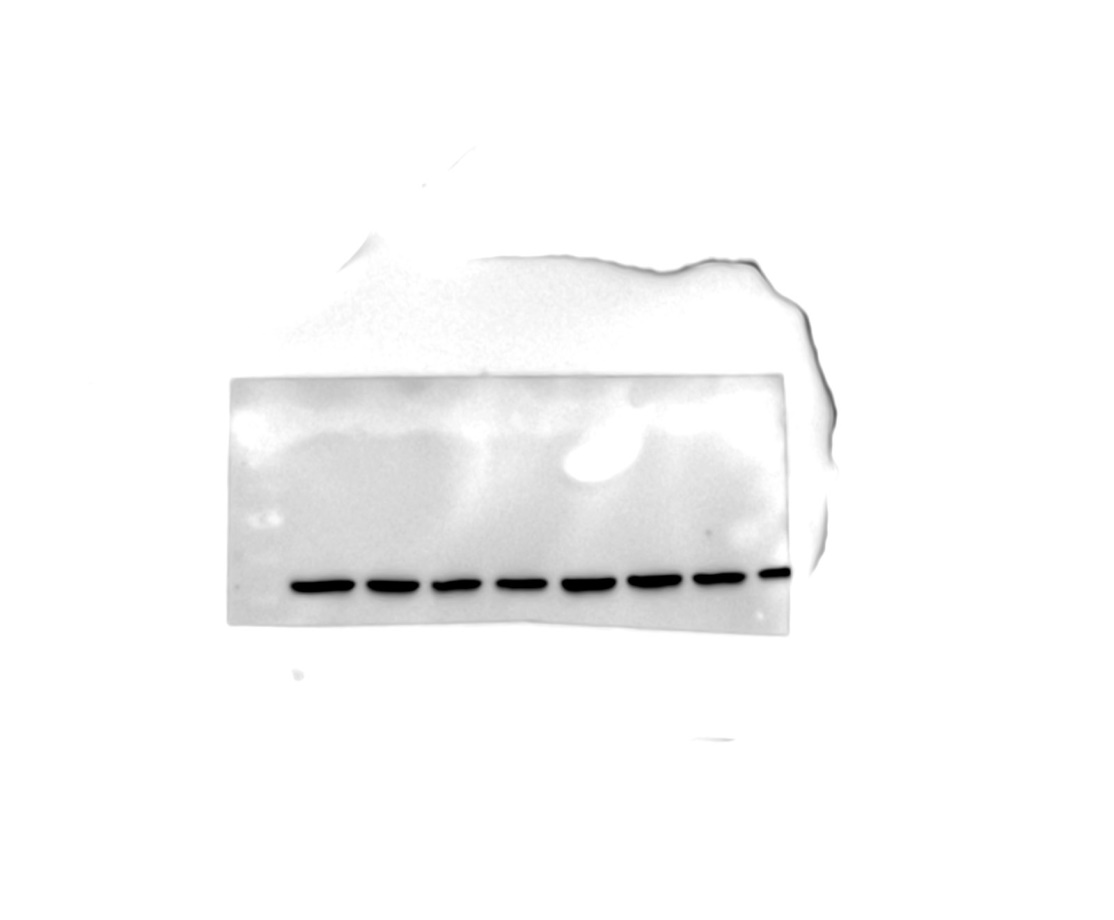


***Rab 7 transfection*** (lane 1, Marker; the other lanes are not included in the study.)

SFTSV NP (lanes 2-5)


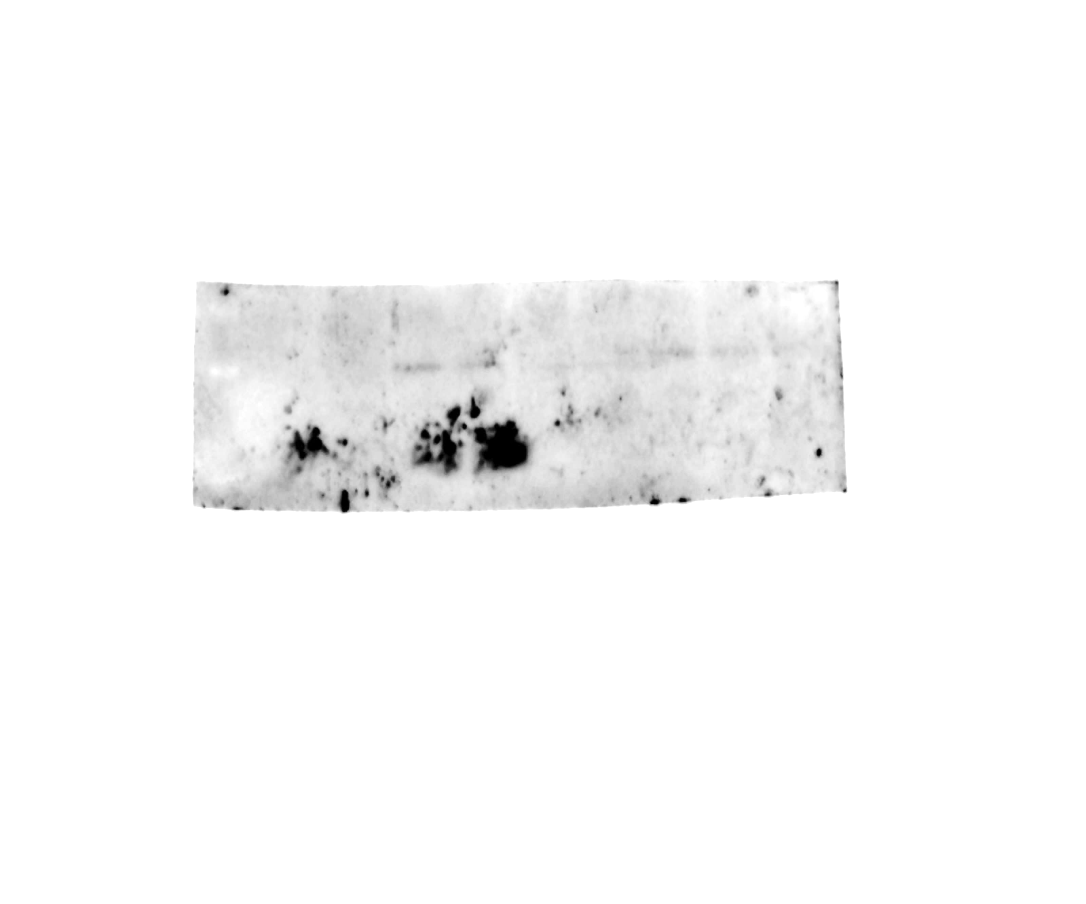


Actin (lanes 2-5)


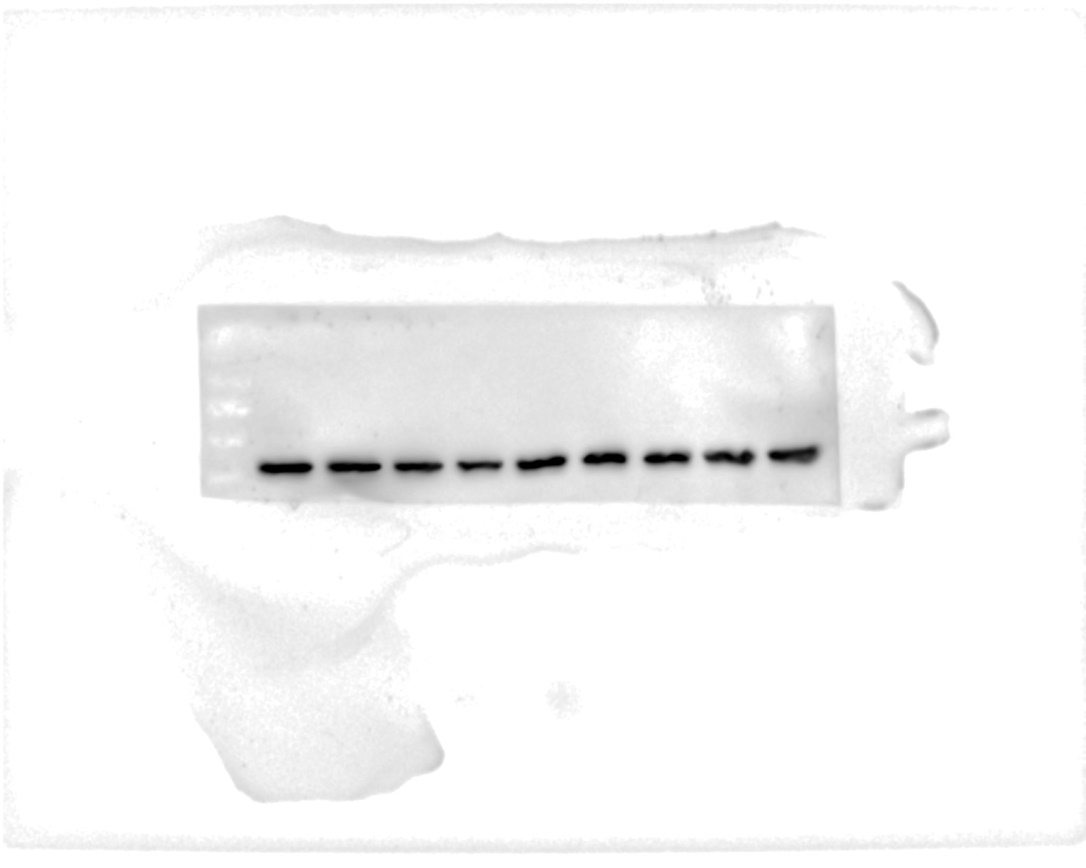


**Fig. 4B**

NP


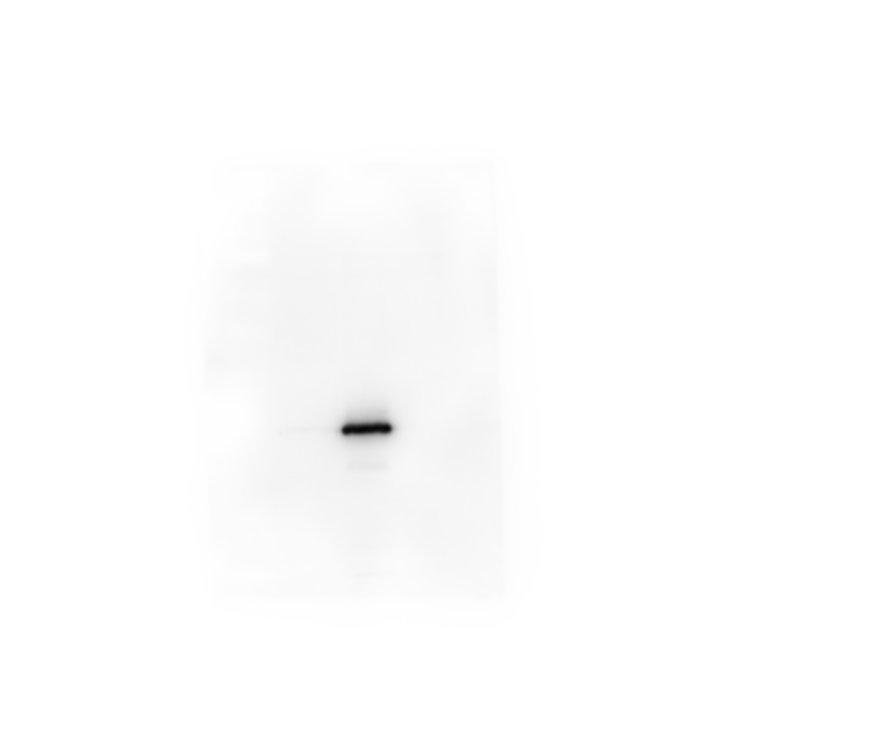


Gn


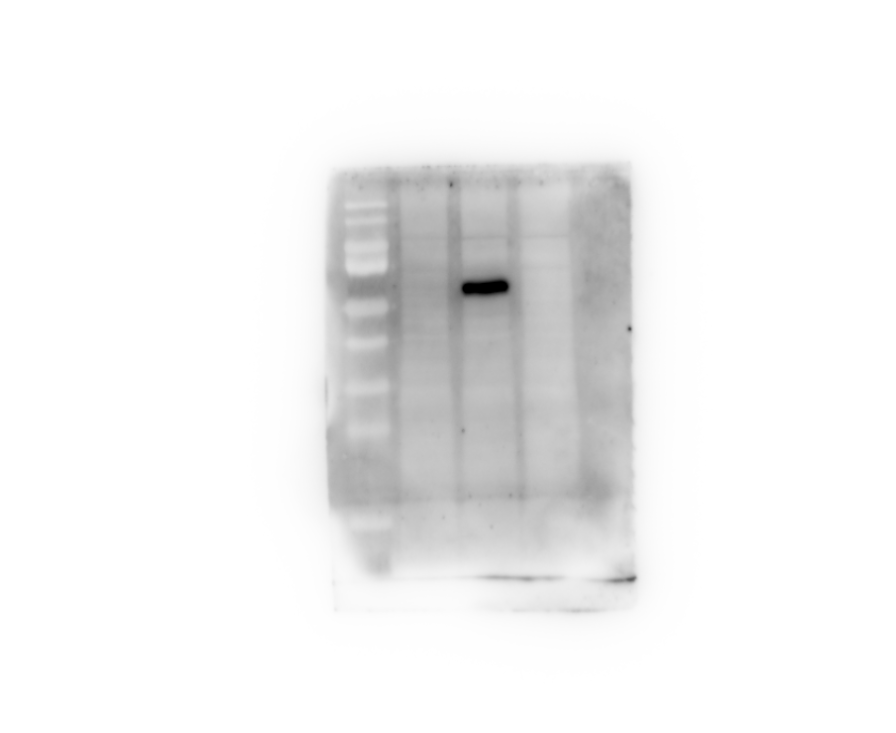


Gc


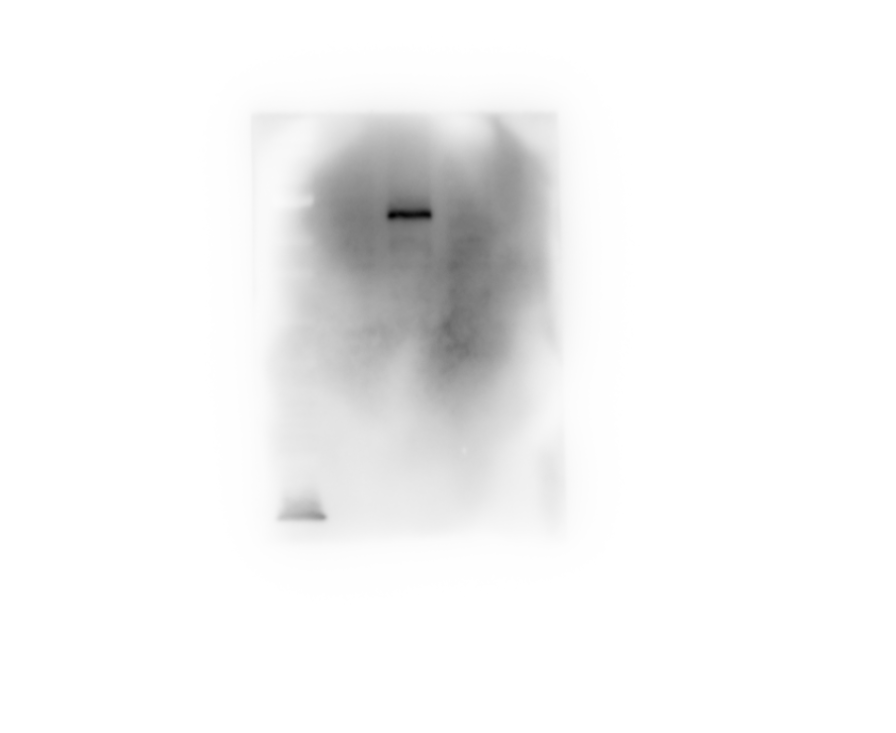


Actin


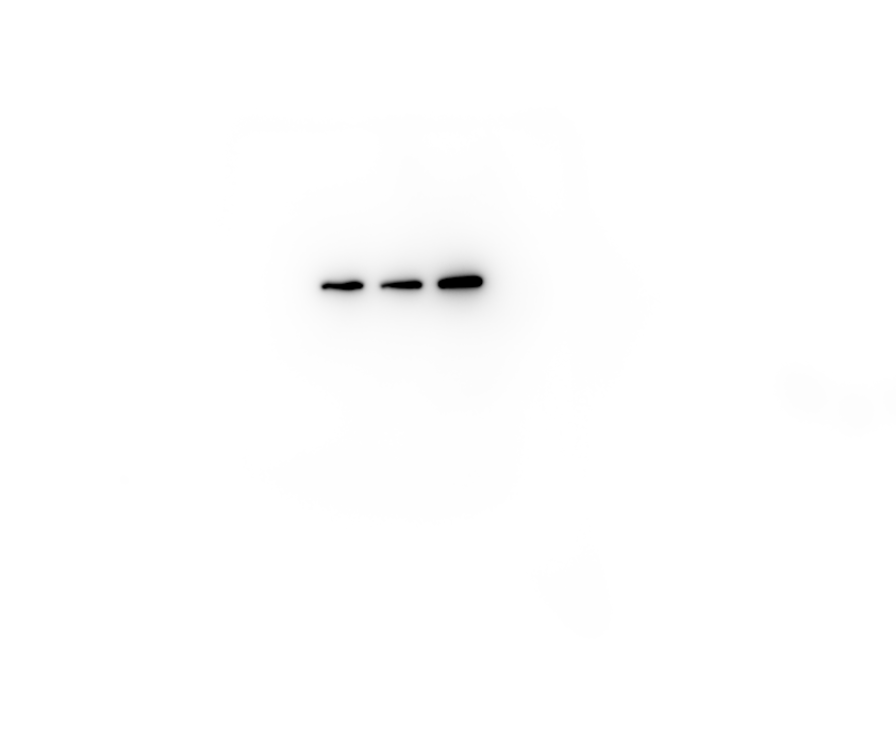

Supplement: Supplementary file 5 — Additional file 5. Original images of Western blots in Fig. 2D, Fig. 3D, Fig. 3E, Fig. 3G, and Fig. 4B, accompanied with brief instructions. [file 12916_2022_2558_MOESM5_ESM.docx]
